# Description of a Remarkable New Skate Species of *Leucoraja* Malm, 1877 (Rajiformes, Rajidae) from the Southwestern Indian Ocean: Introducing 3D Modeling as an Innovative Tool for the Visualization of Clasper Characters

**DOI:** 10.3390/biology13060405

**Published:** 2024-06-02

**Authors:** Simon Weigmann, Matthias F. W. Stehmann, Bernard Séret, Hajime Ishihara

**Affiliations:** 1 Elasmo-Lab, Elasmobranch Research Laboratory, Sophie-Rahel-Jansen-Str. 83, 22609 Hamburg, Germany; 2 Leibniz Institute for the Analysis of Biodiversity Change (LIB), Centre for Taxonomy and Morphology, Zoological Museum, Martin-Luther-King-Platz 3, 20146 Hamburg, Germany; 3 ICHTHYS, Ichthyological Research Laboratory and Consultant, Hildesheimer Weg 13, 22459 Hamburg, Germany; stehmann@ichthys-fisch.info; 4 IchtyoConsult, 6 bis rue du Centre, 91430 Igny, France; seret.bernard@orange.fr; 5 W&I Associates Co., Ltd., 1020-39 Kudencho, Sakae-ku, Yokohama 247-0014, Japan; h.ishihara@wi-associates.co.jp

**Keywords:** Chondrichthyes, Elasmobranchii, rough skates, systematics, taxonomy, diversity, morphology, clasper features, morphometrics, meristics

## Abstract

**Simple Summary:**

Skates of the genus *Leucoraja* Malm, 1877, are small to medium-sized skates that usually have a short, obtusely angled snout. Until now, 14 valid species of this genus have been identified mostly in the Atlantic, but also in the Indian Ocean. In the 1970s and 1980s, a total of eight specimens of an unusual skate species were collected by researchers working on the Madagascar Ridge, an elevated area of seabed in the southwestern Indian Ocean. Despite their long snouts, the specimens could unambiguously be assigned to the genus *Leucoraja* due to the typical features of their claspers. Comparisons with congeners clearly indicated that these remarkable specimens represent a species new to science. It can easily be distinguished from all 14 congeners by the long and acutely pointed snout. Furthermore, it appears to occur only on the Madagascar Ridge, distant from the known distribution areas of all congeners, and shows several unique aspects in its clasper morphology. Due to the importance of the clasper features, 3D modeling is introduced as a new tool for the visualization of clasper characters. The newly described species is named Brown longnose skate.

**Abstract:**

A remarkable new deep-water skate, *Leucoraja longirostris* n. sp., is described based on eight specimens caught during different expeditions to the southern Madagascar Ridge in the southwestern Indian Ocean. The new species differs from all congeners by its remarkably long and acutely angled snout (horizontal preorbital length 17.2–22.6% TL vs. 8.5–11.9% TL and 4.2–6.1 vs. 1.7–3.5 times orbit length, snout angle 65–85° vs. 90–150°). Furthermore, it is apparently endemic to the Madagascar Ridge, distant from the known distribution areas of all congeners. In addition to *L. fullonica* and *L. pristispina*, *L. longirostris* n. sp. is also the only species with plain dorsal coloration. Furthermore, the new species is the only *Leucoraja* species with an external clasper component dike and, besides *L. wallacei*, the only one with four dorsal terminal (dt) cartilages. The shape of the accessory terminal 1 (at1) cartilage with four tips is also unique within the genus. A new approach for the visualization of the clasper characters is introduced based on 3D models of all skeletal and external features. This enables a much easier and much more precise interpretation of every single clasper component, of the entire structure, and, in particular, the relationship between external features and skeletal cartilages. A new English translation of the first diagnosis of *Leucoraja* is provided, along with a revised generic diagnosis and a key to the species of *Leucoraja* in the Indian Ocean.

## 1. Introduction

The family Rajidae de Blainville, 1816 (Chondrichthyes, Elasmobranchii, Rajiformes) is the most speciose batoid family, comprising 18 genera and 158 species (as of 16 April 2024), attaining maximum sizes of 33–264 cm total length (TL) and occurring in 0–3280 m depths of all oceans, with the center of distribution in polar and cool temperate regions (updated from Weigmann [1,2], while considering Last et al. [3,4]). The genus *Leucoraja* was erected by Malm [5] without providing a generic diagnosis. Jordan [6] later designated *L. fullonica* (Linnaeus, 1758) as the type species. The genus was lowered to the subgeneric rank of *Raja* by Stehmann [7], who also gave the first diagnosis of *Leucoraja*. Subsequently, the subgenus was re-elevated to the generic rank by McEachran and Dunn [8]. Its members are characterized by the presence of the external clasper components promontory and roll, as well as several rows of thorns along the median disc and tail, with the mid-row typically smaller than the lateral row(s) to completely reduced in adults, while simultaneously the parallel row thorns become larger [4]. However, due to the large interspecific variation in external characters, the clasper features presently appear to be the most reliable generic characters for the differentiation of *Leucoraja* from the generally similar genus *Rajella* Stehmann, 1970 [9]. Although several studies have called for a reliable phylogenetic hypothesis of skates [10], the monophyly within these two (and further) rajid genera has not yet been examined comprehensively, neither morphologically nor genetically. Several studies from different fields of research have focused on a single species, particularly the model organism *L. erinacea* (Mitchill, 1825) [11,12,13,14], as well as *L. naevus* (Müller and Henle, 1841) [15]. Phylogenetic studies examining *Leucoraja* or *Rajella* species include those by McEachran and Dunn [8], Turan [16], Aschliman et al. [17], Naylor et al. [18,19], Chiquillo et al. [20], and Lynghammar et al. [21]. Nevertheless, none of these studies had the requisite taxonomic representation to comprehensively address the phylogenetic affinities within the respective genus and most studies have focused on relationships within regional groups, thereby hampering broad phylogenetic inferences due to sampling bias and historical and geographic constraints [20].

The 14 currently described species of *Leucoraja* attain maximum sizes of 30–120 cm TL and are found in the Indian and Atlantic oceans, the latter representing the center of the distribution of the genus with 12 of the 14 valid species occurring therein (updated from [1,4]). Of these 12 species, only *L. compagnoi* (Stehmann, 1995) and *L. wallacei* (Hulley, 1970) are also found outside of the Atlantic Ocean, i.e., in continental waters of the southwestern Indian Ocean [1,4,22].

The new species formally described below, *Leucoraja longirostris* n. sp., is the fourth known species of the genus from the western Indian Ocean. It was caught on the southern end of the Madagascar Ridge at Walters Shoals. The other three species, *L. compagnoi*, *L. elaineae*, Ebert and Leslie, 2019, and *L. wallacei*, have only been recorded from the outer continental shelf and upper continental slope. The first specimen of *L. longirostris* n. sp., a subadult male, was collected in 1977, another juvenile female in 1979. However, due to the lack of an adult male, the new species could not definitely be assigned to either *Leucoraja* or *Rajella*. Six further specimens of the new species were collected aboard the Russian RV ‘Vityaz’ during its 17th cruise in 1988 and 1989, together with many other new and rare deep-water sharks, skates and rays, and chimaeras. Since these six specimens included an adult male, the generic assignment of the new species became possible.

The description of the new species represents contribution No. 24 to the series “Deep-water chondrichthyan fishes of RV ‘Vityaz’ cruise 17 and other Soviet cruises in the Indian Ocean”, initiated with the description of *Rhinochimaera africana* [23]. A new English translation of the first diagnosis of *Leucoraja* is provided, along with a revised generic diagnosis to take account of the fact that the diagnosis for *Leucoraja* (as a subgenus of *Raja*) was given more than 50 years ago by Stehmann [7] (p. 150) in German and was based only on three northeastern Atlantic species. In the meantime, 11 further species (plus the new species described herein) have been assigned to *Leucoraja* or have been newly described and cover a wide range of morphotypes and distribution areas. A key to the species of *Leucoraja* in the Indian Ocean is also given.

## 2. Materials and Methods

Institutional acronyms follow Fricke and Eschmeyer [24]. External morphometric measurements were taken by vernier caliper to one tenth of a millimeter (mm) from the RV ‘Vityaz’ specimens prior to preservation and from the other two specimens preserved in 70% ethanol or 4% formaldehyde. Measurements were taken between perpendicular lines where relevant and largely following Bigelow and Schroeder [25]. Exceptions: prenasal snout length from snout tip to transverse line through anterior edge of nostrils, and orbit plus spiracle length after Clark [26]; ventral head length from snout tip to transverse line through fifth gill slits after Ishiyama [27]; tail and nasal curtain measurements after Hubbs and Ishiyama [28]; spiracle length measured diagonally as aperture proper; length of pelvic lobes measured from the point of articulation of the anterior lobe according to Stehmann [29]. Terminology of clasper glans components and skeleton cartilages follows Hulley [30,31], Stehmann [7], and Weigmann et al. [9]. Skeletal morphometric measurements of the cranium and scapulocoracoid were made according to McEachran and Compagno [32] and of pelvic girdle according to Stehmann et al. [33]; the vertebral counts were carried out according to Springer and Garrick [34] and Krefft [35]. Skeletal morphometric measurements and meristics were taken and counted from radiographs, except for those of the scapulocoracoid which were taken from the dissected element. The following description considers field notes and color photographs of the fresh and 70% ethanol-preserved specimens from RV ‘Vityaz’.

Every single external component and internal cartilage of the left clasper was dissected and all cartilages were cleaned step by step. Afterwards, 3D models were created on the computer for each single component using the software Blender 2.81. These were then assembled again to develop a faithful copy of the original clasper. During the modeling process, the original claspers and their cartilages were considered, as were photographs of the fresh and preserved claspers, radiographs of the preserved claspers, and drawings of the left clasper and its cartilages.

The catch locations of the type specimens of the new species are shown in a map, which was generated based on the Global Relief Model ETOPO1 by the NOAA, the National Oceanic and Atmospheric Administration [36]. Country borders, lakes, and rivers were visualized by means of the shapefiles supplied by ESRI for the ArcExplorer-Java Edition for Education 2.3.2 (AEJEE). For a map with all stations of cruise 17 of RV ‘Vityaz’, see, for example, Weigmann et al. [37].

The holotype and five paratypes were deposited in the Zoological Museum Hamburg (ZMH) of the Leibniz Institute for the Analysis of Biodiversity Change (LIB), and one paratype each was deposited at the Tokyo University of Marine Science and Technology, Museum of Fishery Sciences, Tokyo (MTUF; formerly deposited at the National Research Institute of Far Seas Fisheries, Fisheries Research Agency: FSFRL) and the Zoological Museum of Moscow University (ZMMU).

*Comparative material examined*: ***Leucoraja circularis*** (Couch, 1838): ZMH 101019 (ex ISH 17-1960), 2 females, 610 mm and 700 mm TL, RV ‘Anton Dohrn’, northern North Sea, 61°08′ N, 03°10′ E, 350 m depth, bottom trawl, 1 April 1960; ZMH 101178 (ex ISH 198-1960), adult male, 785 mm TL, RV ‘Anton Dohrn’, northern North Sea, 60°13′ N, 03°22′ E, 160–200 m depth, bottom trawl, 11 December 1960; ZMH 123445 (ex ISH 22-1990), adult female, 877 mm TL, RV ‘Walther Herwig’, station 136/hole 48/90, North Sea, Halibut Bank, 61°13′ N, 0°37′ E, 163–164 m depth, 12 February 1990; ***Leucoraja compagnoi***: ZISP 48406, holotype, juvenile female, 292 mm TL, V ‘POLTAVA’, station 443, off South Africa, 30°44′ S, 15°18′ E, 550 m depth, 28 January 1970; ***Leucoraja elaineae*** (detailed data and images kindly provided by David A. Ebert): SAIAB 13742 (PCH 80–20), holotype, female, 330 mm TL fresh (318 mm TL after preservation), RV ‘Fridtjof Nansen’, off Malindi, Kenya, 03°25′ S, 40°23′ E, 484 m depth, bottom trawl, 11 December 1980; ***Leucoraja erinacea***: ZMH 119935 (ex-ISH 3690-1979), 3 adult males, 455, 465, and 482 mm TL, RV ‘Anton Dohrn’, station 6432/79, off New York, 40°04.8′ N, 72°07.5′ W, 78 m depth, 140′ bottom trawl, 12 November 1979; ***Leucoraja fullonica***: ZMH 100415 (ex-ISH 18-1956), juvenile male, 587 mm TL, RV ‘Anton Dohrn’, station 677/56, west of southern Norway, 61°20′ N, 02°18′ E, 160–170 m depth, bottom trawl, 26 February 1956; ZMH 100435 (ex-ISH 31-1956), juvenile female, 532 mm TL, RV ‘Anton Dohrn’, station 702/56, west of Shetland Islands, 60°06′ N, 03°13′ W, 155 m depth, bottom trawl, 6 March 1956; ZMH 100812 (ex-ISH 158-1959), juvenile female, 560 mm TL, RV ‘Anton Dohrn’, station 3276/59, Faroe Canal, 61°15′ N, 01°17′ W, 180–190 m depth, bottom trawl, 9 May 1959; ZMH 101817 (ex ISH 72-1964), presumably adult or subadult female, 762 mm TL, RV ‘Anton Dohrn’, station 23/64, Faroe Bank, 60°47′ N, 09°22′ W, 180 m depth, 140′ bottom trawl, 26 January 1964; ZMH 107576 (ex ISH 5-1969), 2 juvenile females, 580 and 656 mm TL, RV ‘Anton Dohrn’, station 588/69, west of Shetland Islands, 60°51′ N, 02°21′ W, 190 m depth, 140′ bottom trawl, 26 July 1969; ***Leucoraja garmani*** (Whitley, 1939): ZMH 119923 (ex-ISH 3535-1979), adult male, 306 mm TL, RV ‘Anton Dohrn’, station 6380/79, off south of Charleston, 32°17′ N, 78°56′ W, 148–156 m depth, 140′ bottom trawl, 1 November 1979; ***Leucoraja leucosticta*** (Stehmann, 1971): ZMH 25255 (ex-ISH 237-1963), paratype, female, 569 mm TL, Guinean Trawling Survey 1, trans. 3, station 6, off Conakry, Gulf of Guinea, 10°49′ N, 17°0′ W, 100 m depth, bottom trawl, 8 December 1963; ***Leucoraja melitensis*** (Clark, 1926): ZMH 114555 (ex-ISH 806-1976), pair of claspers from an adult male, 450 mm TL, 320 mm DW, off the coast of Tunisia; ***Leucoraja naevus***: ZMH 101540 (ex-ISH 9-1963), subadult male, 533 mm, TL and juvenile male, 170 mm TL; northern North Sea, off eastern Orkneys, 58°49′ N, 01°35′ W, 106 m depth, bottom trawl, 30 January 1963; ZMH 123443 (ex-ISH 11-1990), female, 574 mm TL, and adult male (not found), 608 mm TL, RV ‘Walther Herwig’, station 115/90, Montrose Bank, 56°45′ N, 01°18′ W, 69–70 m depth, 7 February 1990; ***Leucoraja ocellata*** (Mitchill, 1815): ZMH 103088 (ex-ISH 7-1965), female, 545 mm TL, RV ‘Walther Herwig’, station 25/65, George Bank, 41°55′ N, 66°29′ W, 80 m depth, bottom trawl, 30 January 1965; ZMH 107707 (ex-ISH 61-1970), juvenile female, 430 mm TL and juvenile male, 510 mm TL, RV ‘Walther Herwig’, station 367/70, 41°40′ N, 66°20′ W, 90 m depth, 200′ bottom trawl, 22 July 1970; ZMH 107717 (ex-ISH 62-1970), juvenile female, 345 mm TL, and 2 females, ~650 and ~700 mm TL, RV ‘Walther Herwig’, station 366/70, 41°29′ N, 66°37′ W, 85 m depth, 200′ bottom trawl, 22 July 1970; ZMH 107741 (ex-ISH 57-1970), female, 780 mm TL, RV ‘Walther Herwig’, station 490/70, 40°43′ N, 67°49′ W, 76–80 m depth, 200′ bottom trawl, 23 August 1970; ZMH 112182 (ex-ISH 1195-1974), adult male, 800 mm TL, and juvenile male, RV ‘Walther Herwig’, station 98/74, 42°08′ N, 67°10′ W, 54–78 m depth, 180′ herring bottom trawl, 30 March 1974; ***Leucoraja pristispina*** Last, Stehmann and Séret, 2008 (detailed morphometrics kindly provided by Peter R. Last): CSIRO CA 3905, holotype, adult male, 362 mm TL, southwest of Imperieuse Reef (Rowley Shoals), Western Australia, 18°07′ S, 118°09′ E, 400–404 m depth, 5 February 1983; CSIRO CA 4339, paratype, female, 360 mm TL; CSIRO CA 4342, paratype, female, 378 mm TL, southwest of Imperieuse Reef, Western Australia, 17°45′ S, 118°30′ E, 442–460 m depth, 5 February 1983; CSIRO CA 4370, paratype, adult male, 374 mm TL, northeast of Rowley Shoals, Western Australia, 15°42′ S, 120°34′ E, 500–504 m depth, 10 February 1984; CSIRO CA 4403, paratype, adult male, 348 mm TL, southeast of Scott Reef, west of Bonaparte Archipelago, Western Australia, 14°12′ S, 122°32′ E, 348–350 m depth, 14 February 1984; CSIRO H 2026–1, paratype, female, 372 mm TL, northeast of Mermaid Reef, Rowley Shoals, Western Australia, 16°59′ S, 120°13′ E, 396 m depth, 12 April 1989.

Nomenclatural Acts: the electronic edition of this article conforms to the requirements of the amended International Code of Zoological Nomenclature, and hence the new names contained herein are available under that code from the electronic edition of this article. This published work and the nomenclatural acts it contains have been registered in ZooBank, the online registration system for the ICZN. The ZooBank LSIDs (Life Science Identifiers) can be resolved and the associated information viewed through any standard web browser by appending the LSID to the prefix “https://zoobank.org/” (accessed on 22 May 2024). The LSID for this publication is urn:lsid:zoobank.org:pub:16AAEF2A-529F-417C-8810-FEB0A77D5B96. The electronic edition of this work was published in a journal with an ISSN, and has been archived and is available from the following digital repositories: PubMed Central, LOCKSS.

## 3. Results

**Systematic account**

Class Chondrichthyes;

Subclass Elasmobranchii;

Order Rajiformes;

Family Rajidae de Blainville, 1816.

### 3.1. Genus Leucoraja Malm, 1877

Type species *Raja fullonica* Linnaeus, 1758 by subsequent designation of Jordan [6].

**Translation of the first diagnosis by Stehmann** [7], **no diagnosis had been given by Malm** [5]; although Stehmann’s [7] diagnosis had already been translated by the Translation Bureau (OK), Foreign Languages Division, Department of the Secretary of State of Canada, Fisheries Research Board of Canada, Biological Station, St. John’s Nfld., Translation Series No. 1745, in 1971, we decided to make a new translation, closer to Stehmann [7] and established taxonomic terminology:

I. Genus *Raja* Linnaeus, 1758

3. Subgenus *Leucoraja* Malm, 1877

Göteborgs och Bohusläns Fauna: 609 (type species: *Raja fullonica* Linnaeus, 1758, by subsequent designation of JORDAN, Stanford Univ. Publs No 39: 391, 1919).

Diagnosis: a subgenus of the genus *Raja* Linnaeus, 1758 with the following characters: clasper glans with external pseudosiphon; components slit, cleft, rhipidion, promontory, roll and spur are present, like also, but not characteristic of the subgenus, shield, sentinel and spike. 3 dT [dorsal terminal] cartilages, 2–3 aT [accessory terminal] cartilages and vT [ventral terminal] in the clasper skeleton; dT_1_, dT_2_, dT_3_, aT_1_ and aT_2_ are of group-specific appearance. Length of rostrum shorter than that of neurocranium, anterior fontanelle elongated egg-shaped and clear cut, posterior fontanelle broader in posterior than anterior part. Disc rhomboid with rounded apices; snout short to moderately long. Disc dorsally largely prickly, ventrally almost smooth; orbital thorns arranged in half rings, mostly a triangle of thorns on nape-shoulder region; median thorn rows at most in juveniles, thorn rows on trunk and tail in 2–4 parallel rows. Dorsal ground color bright or dark, with or without pattern; if with pattern, it is pronounced species-specifically in the form of symmetrical blotching or as ocelli. Ventral side uniformly white. Vtr [trunk vertebrae]: 30–37; Vprd [predorsal tail vertebrae]: 62–81; pectoral radials: 76–91. Species in NE-Atlantic: *fullonica*, *circularis*, *naevus*. Presumably affiliated or closely related species outside the NE-Atlantic: *melitensis* (Mediterranean Sea). Distribution: longitudinally eastern Atlantic, Mediterranean.

**Revised generic diagnosis of *Leucoraja* Malm, 1877** (based on 15 species known to date, including the new species described herein). Members of this rajid skate genus can be found in the Atlantic and Indian Oceans on shelves and slopes in temperate and tropical latitudes. They are very small (total length to about 30 cm) or small (to about 60 cm) to moderately large (up to about 120 cm). Disc usually rhombic to inverse heart-shaped with short, obtusely angled to moderately long and pointed snout, but snout long and acutely angled in the new species. Tail solid and gradually tapering to tip. Tail longer than distance from snout tip to mid-vent, tail length 1.0–1.6 times precloacal length. Bases of dorsal fins confluent or with short interspace. Postdorsal tail section very short. Sensory and mucus pores not black-marked. Upper sides of disc and tail usually largely covered with dermal denticles. Most species with more or less pronounced thorn pattern including thorns on snout, half rings on orbital rims, a triangular patch on nape-shoulder region, a median row from trunk to first dorsal fin (reduced with advancing age in several species), and 2–6, mostly 2–4 parallel rows of large thorns. The triangular patch of thorns on nape-shoulder region usually increases in size with advancing age, whereas the half rings of thorns on orbital rims are reduced with growth in few species. Interdorsal thorns mostly absent, but 1 to rarely 4 present in some species. Dorsal ground color mostly light to dark brown or gray, may be uniformly colored or with different patterning mostly consisting of spots or blotches, which are either irregularly scattered or arranged in rosettes, in three species the pattern instead consists of two ocelli. Tail with dark cross-bands in some species. Ventral surface whitish or yellowish with irregular grayish or brownish blotches or fin margins in several species. Claspers of adult males rather short, hardly reaching half-length of tail, slender to more massive, with terminal region usually somewhat widened. Components cleft (=pocket *sensu* McEachran [38]), rhipidion, sentinel, shield, slit and spike present; the components promontory and roll are always present and distinguish species of *Leucoraja* from those of the generally similar genus *Rajella*, species of which lack those two components. Claspers partially also with components dike, eperon, pent, pseudosiphon, sentina, and spur. Additionally to axial, dorsal and ventral marginal cartilages, terminal clasper skeleton with 2 accessory terminal cartilages, 3–4 dorsal terminals, and a ventral terminal; dorsal terminal 2 without long, solid distal process. Length of rostrum (rostral shaft) variable but shorter than length of neurocranium (nasobasal length) in all species except for the new species, cranial orbit contour from deeply concave to shallower trapezoid-shaped. Scapulocoracoid with one anterior fenestra without anterior bridge, one postdorsal, and one postventral fenestra. Pelvic girdle straight, posterior contour weakly concave, prepelvic processes massive and short. Upper jaw tooth rows: 30–110; trunk vertebrae (Vtr): 21–37; predorsal tail vertebrae (Vprd): 51–81; pectoral radials: 61–91. Due to the large interspecific variation in external characters, clasper features, in particular the presence of external components promontory and roll, presently appear to be the most reliable generic characters. A list of all 15 valid species of *Leucoraja* can be found under Remarks.

### 3.2. Leucoraja longirostris n. sp.

The species is registered in ZooBank under urn:lsid:zoobank.org:act:04E52A25-837E-4016-97C4-C427B8FBAAA4.

English name: Brown longnose skate

Figure 1, Figure 2, Figure 3, Figure 4, Figure 5, Figure 6, Figure 7, Figure 8, Figure 9, Figure 10, Figure 11, Figure 12, Figure 13, Figure 14, Figure 15, Figure 16, Figure 17, Figure 18, Figure 19, Figure 20, Figure 21, Figure 22, Figure 23, Figure 24, Figure 25, Figure 26, Figure 27, Figure 28, Figure 29, Figure 30, Figure 31, Figure 32 and Figure 33; Table 1, Table 2, Table 3, Table 4 and Table 5

*Leucoraja* sp.―Weigmann 2016: 948, 949 [1].

Holotype **ZMH 26260**, adult male, 711 mm TL, RV ‘Vityaz’, cruise 17, station 2668, Walters Shoals, 33°01.2′ S, 44°36.8′ E–33°05.2′ S, 44°39.2′ E, 1010 m depth, 19.4 m shrimp trawl, trawl # 48, on the bottom for 61 min, 8 December 1988.

Paratypes (7) **ZMH 26261**, juvenile male, 477 mm TL, same data as holotype ZMH 26260; **ZMH 26262**, juvenile male, 381 mm TL, RV ‘Vityaz’, cruise 17, station 2733, Walters Shoals, 33°23.8′ S, 44°06.9′ E–33°21.1′ S, 44°05.6′ E, 750–775 m depth, 29 m shrimp trawl, trawl # 66, on the bottom for 60 min, 18 December 1988; **ZMH 26263**, adult female, 700 mm TL, RV ‘Vityaz’, cruise 17, station 2735, Walters Shoals, 33°36′ S, 44°32′ E–33°38′ S, 44°34′ E, 930–950 m depth, 29 m shrimp trawl, trawl # 68, on the bottom for 75 min, 19 December 1988; **ZMH 26264**, juvenile female, 340 mm TL, same data as paratype ZMH 26263; **ZMH 26265**, juvenile female, 385 mm TL, RV ‘Vityaz’, cruise 17, station 2736, Walters Shoals, 33°58.1′ S, 45°01′ E–33°57′ S, 45°02.5′ E, 1030–1050 m depth, 29 m shrimp trawl, trawl # 69, on the bottom for 47 min, 19 December 1988; **MTUF 30754 (formerly FSFRL EL-545)**, early subadult male, 592 mm TL, 33°34′S, 44°32′E, 956 m depth, bottom trawl, 20 June 1977; **ZMMU P-17803**, juvenile female, 276 mm TL, RV ‘Professor Mesyatsev’, cruise 7, 33°42′ S, 44°39′ E, 920–940 m depth, bottom trawl, trawl # 139, 25 June 1979, collector Dr. A. Kotlyar.

**Diagnosis.** A medium-sized species of the genus *Leucoraja* with disc rhombic to (in adult male) evenly inverse heart-shaped, with broadly rounded outer corners and with body length to mid-vent about equal to tail length from mid-vent. Preorbital snout length 17.2–22.3% TL and distance between first gill slits 11.6–12.9% of TL. Orbits moderately large, horizontal diameter 1.0–1.2 times interorbital width. Dorsal disc largely devoid of denticles, ventrally spinules at least on snout tip. Thorn pattern pronounced with ~5–20 rostral thorns, a half ring of 3–5 thorns on each orbital rim, 1 supraspiracular and 1 interspiracular thorn per side, 3–5 median nuchal thorns, 0–3 lateral nuchal thorns per side, 1–2 suprascapular thorns, 0–2 lateral scapular thorns, 2–3 scapular thorns per side, a median row of 34–41 thorns from behind the shoulder girdle to just before the origin of the first dorsal fin, no interdorsal thorns, and 2–4 parallel rows of thorns on tail. When fresh, dorsal surface plain medium-brown, with grayish mucus coverage; ventral side plain grayish on disc and pelvic fins, snout dusky grayish, sensory pores not marked dark. Bases of nearly equal-sized and long but low dorsal fins confluent or with short interspace of maximally 0.8% TL. Postdorsal tail section short, 2.5–3.8% TL, with low epichordal caudal lobe, which is confluent with the second dorsal fin. Brownish lateral tail folds along posterior 58.1–72.0% of tail. Upper jaw tooth rows 41–49, pectoral-fin radials 69–73, pelvic-fin radials 3 + 15–3 + 18. Clasper lacking external pseudosiphon; inner dorsal lobe with components roll, promontory, slit, and two clefts; inner ventral lobe with components shield, rhipidion, pent, sentinel, spike, and dike. Terminal clasper skeleton with four dorsal terminal cartilages, a ventral terminal, and two accessory terminal cartilages, plus a tiny separate spindle-shaped fibrocartilage between distal tips of dorsal terminal 1 and ventral terminal. Anterior cranial fontanelle with clear-cut contour all around and extending only about one fifth into rostral shaft length. Scapulocoracoid subquadratic, rear corner poorly developed, large oval anterior fenestra without anterior bridge, one moderately large, oval postdorsal and postventral fenestra, respectively. Pelvic girdle with massive ischiopubic bar with nearly straight anterior and deeply concave posterior contour; prepelvic processes short, solid, conical, and somewhat inclined outwards, their length 1.7–2.9 times median thickness of ischiopubic bar. The new species differs from all congeners in the remarkably long and acutely angled snout (horizontal preorbital length 17.2–22.6% TL vs. 8.5–11.9% TL and 4.2–6.1 vs. 1.7–3.5 times orbit length, snout angle 65–85° vs. 90–150°). The new species is apparently endemic to the Madagascar Ridge, distant from the known distribution areas of all congeners by more than 1000 km. In addition to *L. fullonica* and *L. pristispina*, *L. longirostris* n. sp. is also the only species with plain dorsal coloration. Furthermore, the new species is the only *Leucoraja* species with external clasper component dike and, besides *L. wallacei*, the only one with four dorsal terminal cartilages. The shape of the accessory terminal 1 cartilage with four tips is also unique within the genus.

**Description of the holotype.** Values of the paratypes in parentheses, more complex differences are described separately. Where relevant, ratios are based on horizontal measurements unless otherwise stated. Detailed morphometric measurements and meristics are given in Table 1.

**Table 1:** *Leucoraja longirostris* n. sp., morphometrics and meristics. Individual values for the adult male holotype (ZMH 26260), adult female paratype (ZMH 26263), and early subadult male paratype (MTUF 30754), ranges for all juvenile paratypes (*n* = 5), as well as means and standard deviations (SD) for all eight type specimens. Proportional values are expressed as percentages of total length (TL) except for the minimum, maximum, and mean of TL in millimeters.

	Holotype, ZMH 26260, Adult Male		Paratype, ZMH 26263, Adult Female		Paratype, MTUF 30754, Early Subadult Male		Minimum (*n* = 5)	Maximum (*n* = 5)	Mean (*n* = 8)	SD
	mm	% TL	mm	% TL	mm	% TL	% TL	% TL	% TL	
total length (TL)	711.0	100.0	700.0	100.0	592.0	100.0	276.0	477.0	482.8	
disc, width	382.0	53.7	388.0	55.4	315.0	53.2	53.0	54.5	54.0	0.8
disc, length	405.0	57.0	395.0	56.4	335.0	56.6	49.5	54.3	54.3	2.5
snout length, preorbital	153.5	21.6	161.0	23.0	132.0	22.3	17.2	20.7	20.6	1.8
orbit, horizontal diameter	25.6	3.6	26.5	3.8	23.0	3.9	3.5	4.1	3.7	0.2
interorbital width	24.0	3.4	23.8	3.4	20.0	3.4	3.2	3.8	3.5	0.2
spiracle length, slit (opening proper)	18.0	2.5	16.4	2.3	14.0	2.4	2.2	2.6	2.4	0.1
interspiracular width	38.6	5.4	40.6	5.8	32.0	5.4	5.9	6.4	5.9	0.4
orbit + spiracle length	34.4	4.8	30.5	4.4	30.0	5.1	4.2	4.5	4.5	0.3
first dorsal fin (D1), height	19.7	2.8	17.5	2.5	12.0	2.0	1.7	2.4	2.2	0.3
first dorsal fin (D1), base length	46.3	6.5	38.5	5.5	34.0	5.7	6.0	6.8	6.2	0.5
second dorsal fin (D2), height	16.5	2.3	16.6	2.4	12.0	2.0	1.6	2.3	2.1	0.3
second dorsal fin (D2), base length	42.7	6.0	39.5	5.6	32.0	5.4	5.2	6.0	5.7	0.3
tail, postdorsal length	22.5	3.2	19.0	2.7	15.0	2.5	2.6	3.8	3.1	0.5
interdorsal space	0.0	0.0	5.0	0.7	5.0	0.8	0.0	0.0	0.2	0.4
caudal fin (C), base length	22.5	3.2	19.0	2.7	15.0	2.5	2.6	3.8	3.1	0.5
tail, height at pelvic tips	10.4	1.5	11.7	1.7	10.0	1.7	1.5	1.8	1.7	0.1
tail, width at pelvic tips	19.2	2.7	22.3	3.2	18.0	3.0	3.0	3.4	3.1	0.2
tail, height at D1 origin	5.4	0.8	5.3	0.8	5.0	0.8	0.8	0.9	0.8	0.1
tail, width at D1 origin	11.0	1.5	11.5	1.6	8.0	1.4	1.3	1.6	1.4	0.2
tail, lateral fold length	212.0	29.8	182.5	26.1	170.0	28.7	30.8	35.8	31.7	3.4
snout length, preoral	157.0	22.1	166.0	23.7	139.0	23.5	18.4	21.4	21.3	1.8
snout length, prenasal	139.5	19.6	149.0	21.3	123.0	20.8	15.8	19.1	18.8	1.8
head length, ventrally	245.0	34.5	252.0	36.0	208.0	35.1	29.5	33.7	33.1	2.2
mouth width	53.4	7.5	46.7	6.7	47.0	7.9	6.6	7.0	7.0	0.5
internarial width	50.3	7.1	46.3	6.6	43.0	7.3	6.8	7.1	7.0	0.2
nasal curtain, length	33.8	4.8	33.3	4.8	24.0	4.1	3.4	4.4	4.2	0.5
nasal curtain, width each lobe	14.7	2.1	17.3	2.5	11.0	1.9	1.9	2.4	2.1	0.2
nasal curtain, space between lobes	33.7	4.7	20.4	2.9	27.0	4.6	3.9	4.4	4.1	0.6
gill slit length, 1st	8.0	1.1	10.4	1.5	7.0	1.2	1.2	1.4	1.3	0.1
gill slit length, 3rd	9.6	1.4	10.8	1.5	9.0	1.5	1.2	1.6	1.4	0.1
gill slit length, 5th	7.2	1.0	7.0	1.0	6.0	1.0	1.0	1.1	1.0	0.0
interspace first gill slits	87.8	12.3	86.8	12.4	70.0	11.8	11.6	12.9	12.3	0.4
interspace fifth gill slits	51.0	7.2	55.0	7.9	39.0	6.6	7.2	7.8	7.5	0.4
pelvic lobe length from insertion at pelvis, left ant. lobe	78.0	11.0	66.2	9.5	67.0	11.3	10.3	12.4	11.1	0.9
pelvic lobe length from insertion at pelvis, left post. lobe	111.0	15.6	102.4	14.6	93.0	15.7	13.6	15.2	14.8	0.8
pelvic lobe length from insertion at pelvis, right ant. lobe	79.5	11.2	70.0	10.0	67.0	11.3	10.1	11.8	11.1	0.7
pelvic lobe length from insertion at pelvis, right post. lobe	108.0	15.2	100.5	14.4	93.0	15.7	13.7	15.3	14.6	0.7
clasper, postcloacal length	152.0	21.4	-	-	52.0	8.8	8.1	8.4	11.7	6.5
snout tip to mid-cloaca	379.0	53.3	382.0	54.6	306.0	51.7	46.1	50.3	50.5	2.7
mid-cloaca to D1	222.5	31.3	212.5	30.4	194.0	32.8	33.6	37.7	33.8	2.3
mid-cloaca to D2	267.0	37.6	255.6	36.5	232.0	39.2	40.4	43.8	40.2	2.4
mid-cloaca to tail tip	333.0	46.8	314.0	44.9	281.0	47.5	49.7	53.6	49.3	2.8
snout tip to axis max. disc width	253.0	35.6	250.0	35.7	218.0	36.8	31.2	32.7	33.4	2.2
snout angle, °	65.0		71.5		69.0		76.0	85.0	75.3	6.6
pseudobranchial folds left/right	13/13		13/13		13/13		12/12	14/14	12.9/13.0	0.8/0.8
tooth rows, upper jaw	46		41		41		44.0	49.0	44.9	2.9
tooth rows, lower jaw	42		37		39		41.0	46.0	42.4	3.4
trunk vertebrae, Vtr	29		27		30		27.0	30.0	28.6	1.2
predorsal tail vert., Vprd	62		61		61		60.0	64.0	61.8	1.2
total predorsal vert.	91		88		91		88.0	94.0	90.4	2.0
terminal vert., Vterm (ca. in juveniles)	45		46		36		42.0	47.0	43.8	3.5
total vert., Vtotal (ca. in juveniles)	136		134		127		134.0	136.0	134.1	3.0
pectoral radials left/right	70/70		70/70		69/70		69/70	72/73	70.3/70.8	1.2/1.2
pelvic radials left/right	3 + 16/3 + 16		3 + 17/3 + 17		3 + 16/3 + 16		3 + 15/3 + 15	3 + 18/3 + 18	16.5/16.4	1.1/0.9

**Figure 1:**
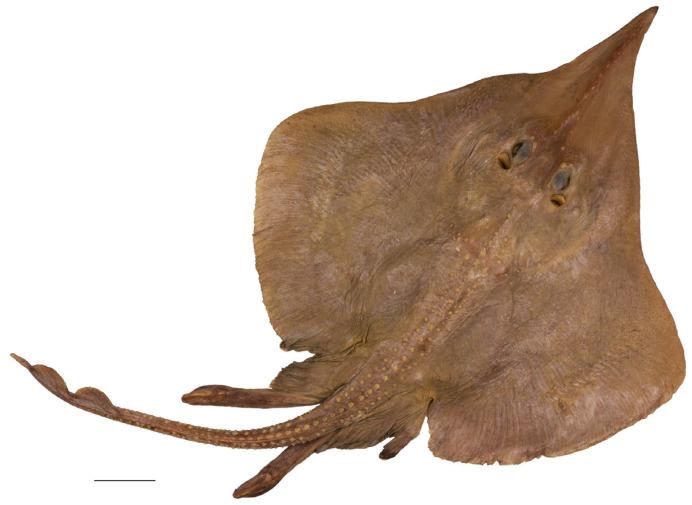
*Leucoraja longirostris* n. sp., ZMH 26260, adult male holotype, 711 mm TL, in total dorsal view. Scale bar: 5 cm.

**Figure 2:**
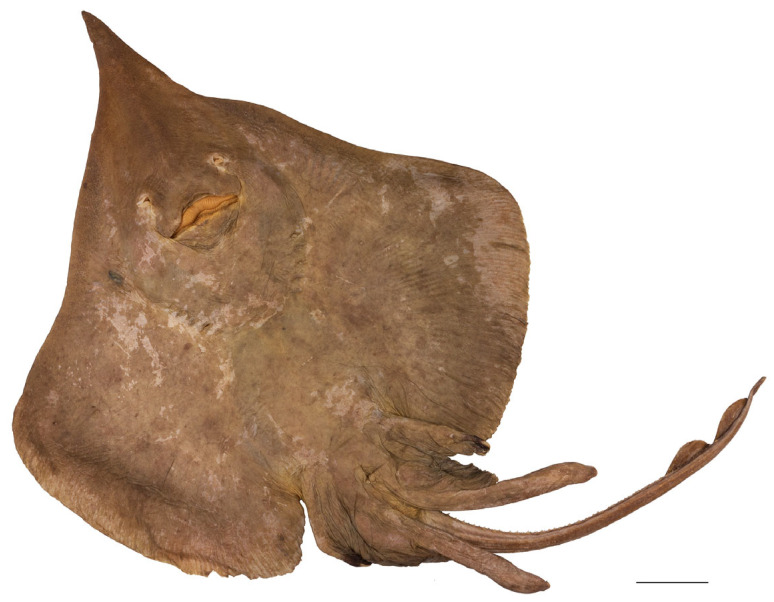
*Leucoraja longirostris* n. sp., ZMH 26260, adult male holotype, 711 mm TL, in total ventral view. Scale bar: 5 cm.

**Figure 3:**
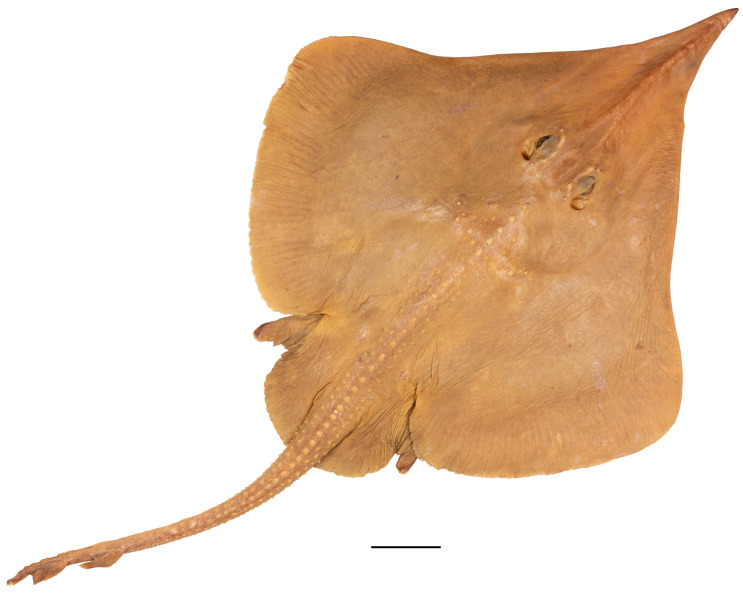
*Leucoraja longirostris* n. sp., ZMH 26263, adult female paratype, 700 mm TL, in total dorsal view. Scale bar: 5 cm.

**Figure 4:**
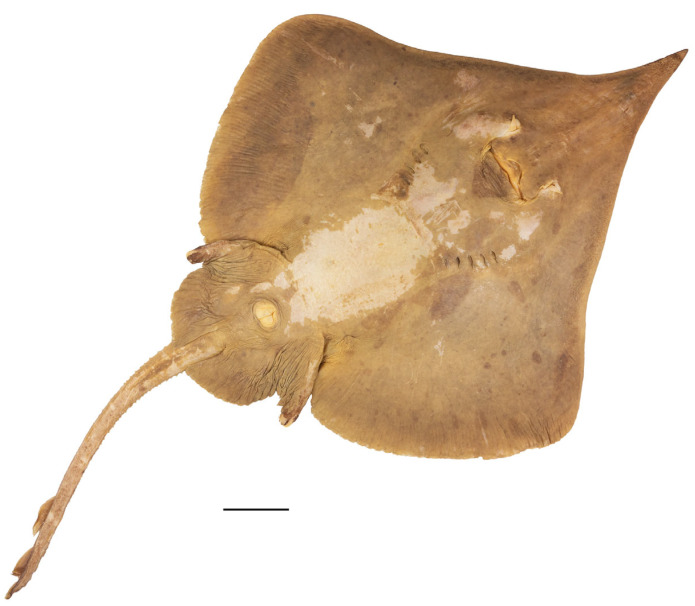
*Leucoraja longirostris* n. sp., ZMH 26263, adult female paratype, 700 mm TL, in total ventral view. Scale bar: 5 cm.

**Figure 5:**
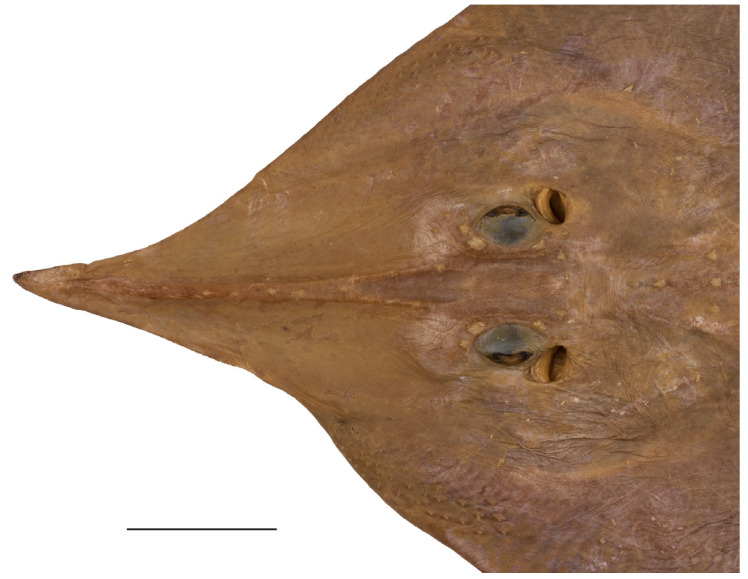
*Leucoraja longirostris* n. sp., ZMH 26260, adult male holotype, 711 mm TL, head in dorsal view. Scale bar: 5 cm.

**Figure 6:**
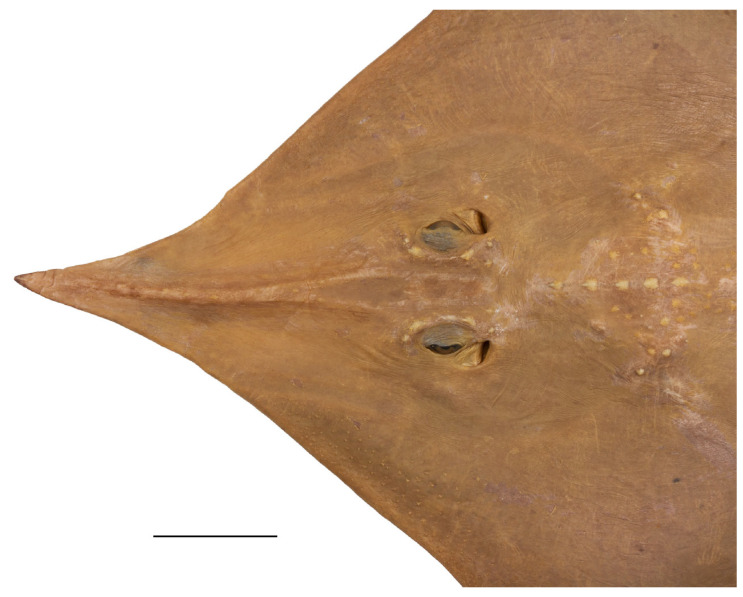
*Leucoraja longirostris* n. sp., ZMH 26263, adult female paratype, 700 mm TL, head in dorsal view. Scale bar: 5 cm.

**Figure 7:**
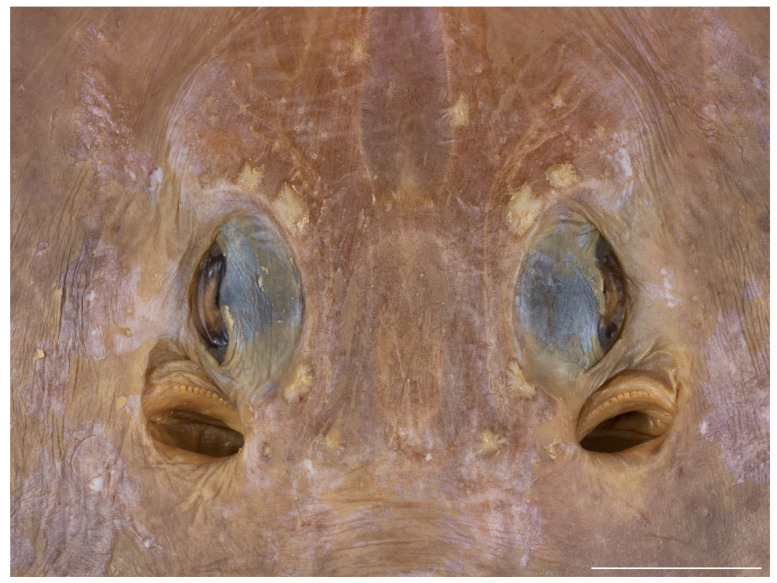
*Leucoraja longirostris* n. sp., ZMH 26260, adult male holotype, 711 mm TL, close-up of orbital and spiracular region. Scale bar: 2 cm.

**Figure 8:**
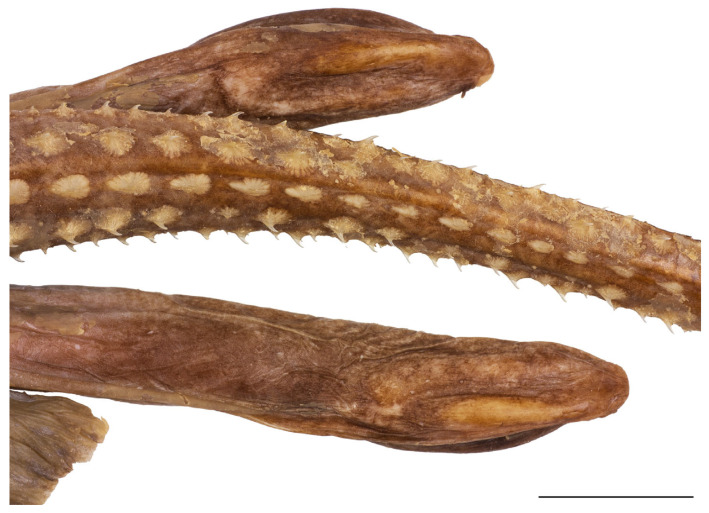
*Leucoraja longirostris* n. sp., ZMH 26260, adult male holotype, 711 mm TL, mid-section of tail in dorsal view. Scale bar: 1 cm. Focus-stacked image.

**Figure 9:**
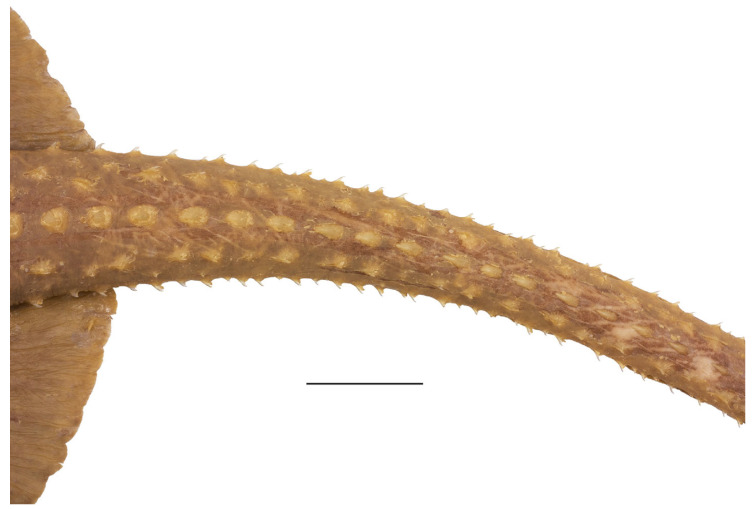
*Leucoraja longirostris* n. sp., ZMH 26263, adult female paratype, 700 mm TL, mid-section of tail in dorsal view. Scale bar: 2 cm.

**Figure 10:**
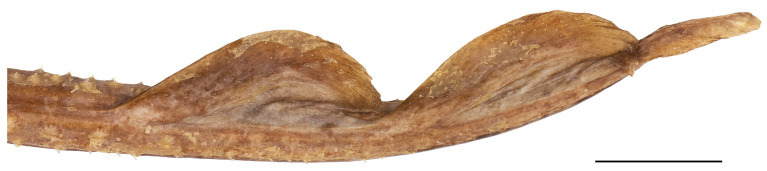
*Leucoraja longirostris* n. sp., ZMH 26260, adult male holotype, 711 mm TL, tail end with dorsal and caudal fins (image reversed). Scale bar: 2 cm.

**Figure 11:**
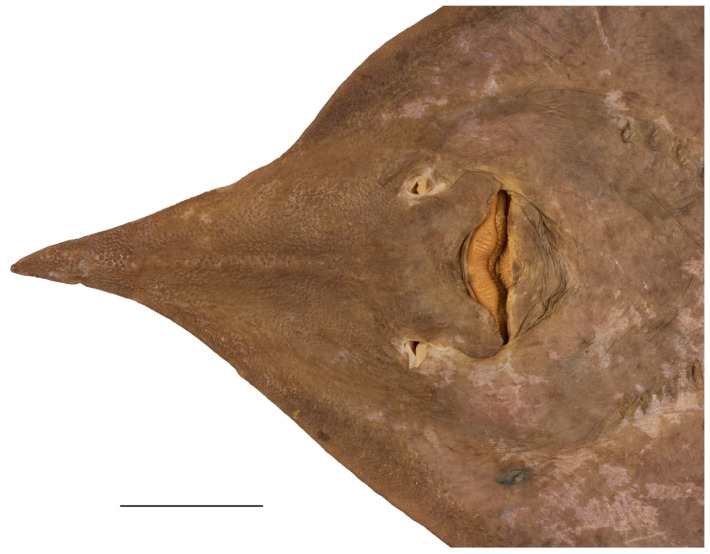
*Leucoraja longirostris* n. sp., ZMH 26260, adult male holotype, 711 mm TL, head in ventral view. Scale bar: 5 cm.

**Figure 12:**
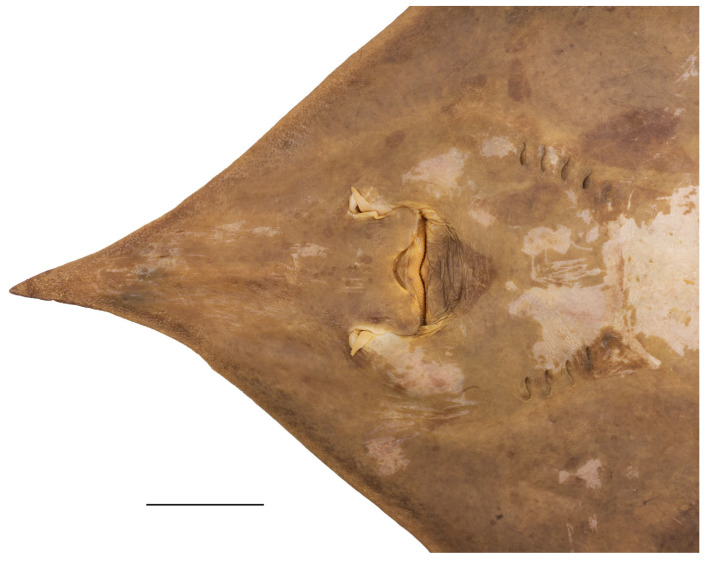
*Leucoraja longirostris* n. sp., ZMH 26263, adult female paratype, 700 mm TL, head in ventral view. Scale bar: 5 cm.

**Figure 13:**
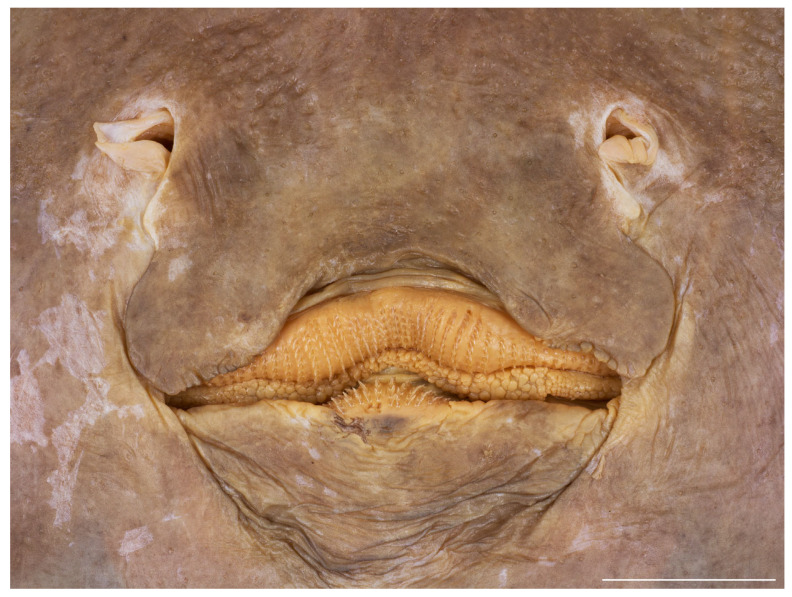
*Leucoraja longirostris* n. sp., ZMH 26260, adult male holotype, 711 mm TL, close-up of mouth–nasal region. Scale bar: 2 cm.

**Figure 14:**
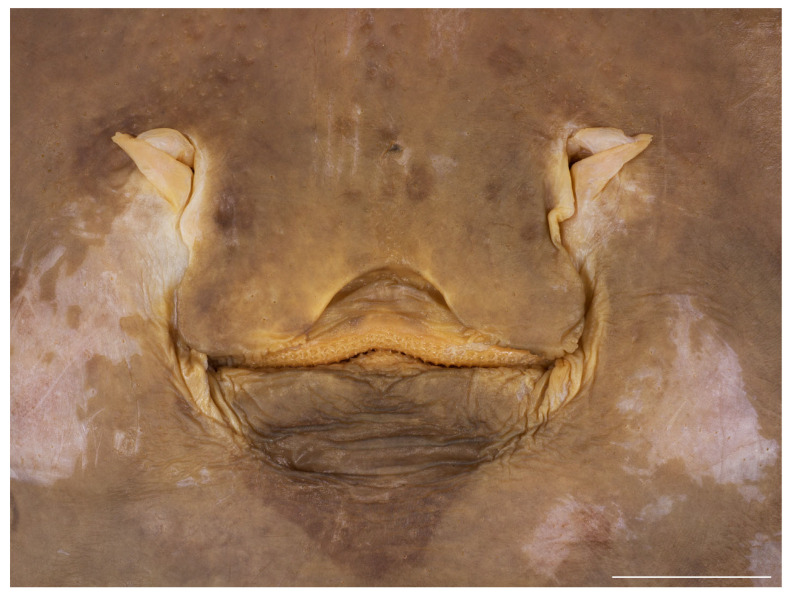
*Leucoraja longirostris* n. sp., ZMH 26263, adult female paratype, 700 mm TL, close-up of mouth–nasal region. Scale bar: 2 cm.

**Figure 15:**
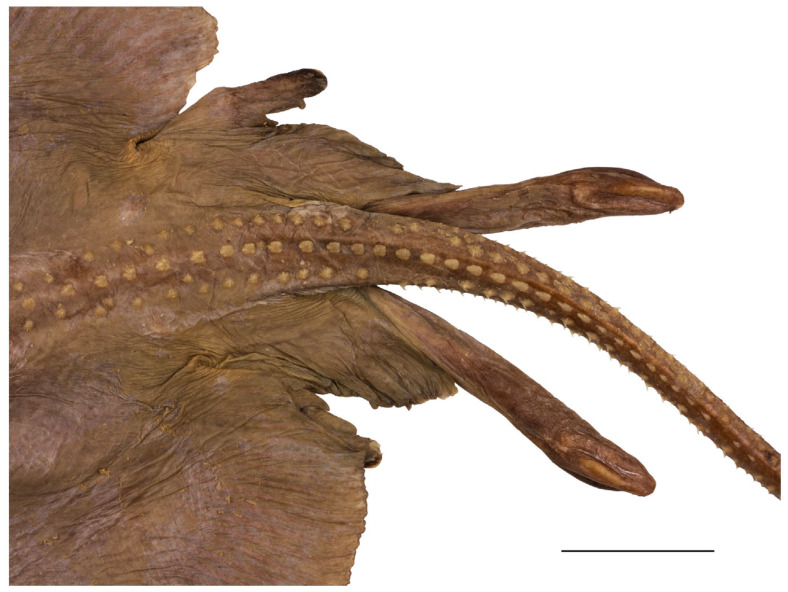
*Leucoraja longirostris* n. sp., ZMH 26260, adult male holotype, 711 mm TL, pelvic region with tail origin and pair of claspers in dorsal view. Scale bar: 5 cm.

**Figure 16:**
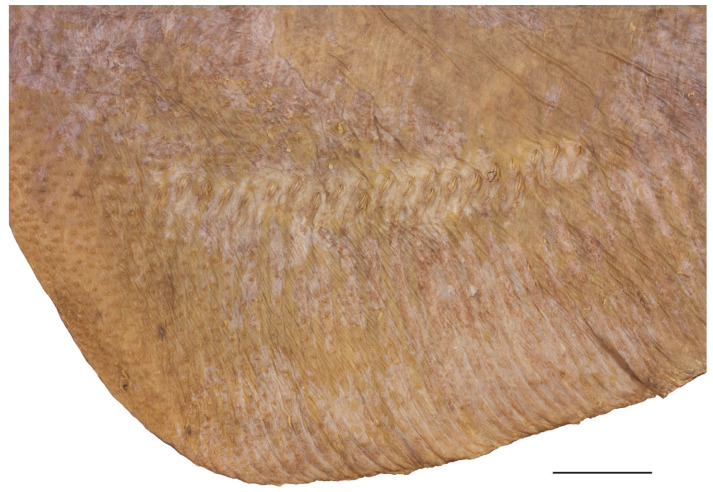
*Leucoraja longirostris* n. sp., ZMH 26260, adult male holotype, 711 mm TL, left field of alar thorns. Scale bar: 2 cm.

**Figure 17:**
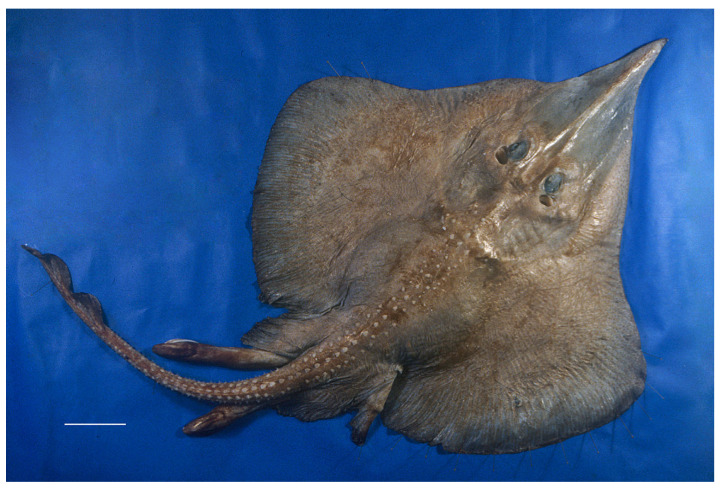
*Leucoraja longirostris* n. sp., ZMH 26260, adult male holotype, 711 mm TL, in total dorsal view taken directly after being caught by MFWS. Scale bar: 5 cm.

**Figure 18:**
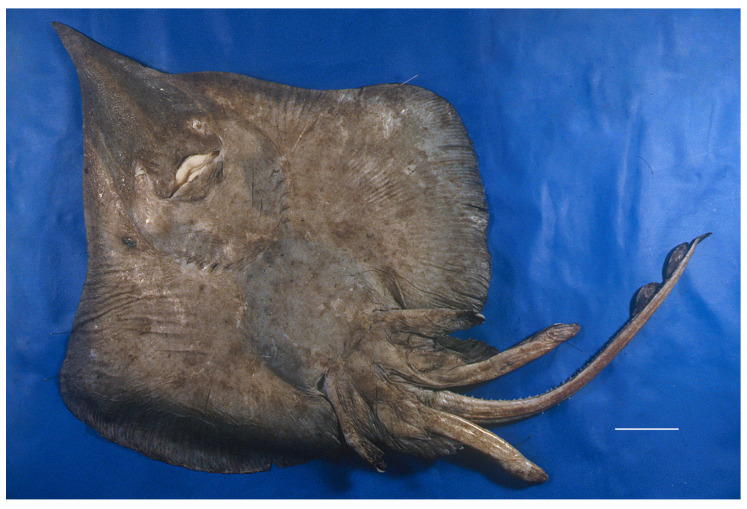
*Leucoraja longirostris* n. sp., ZMH 26260, adult male holotype, 711 mm TL, in total ventral view taken directly after being caught by MFWS. Scale bar: 5 cm.

**Figure 19:**
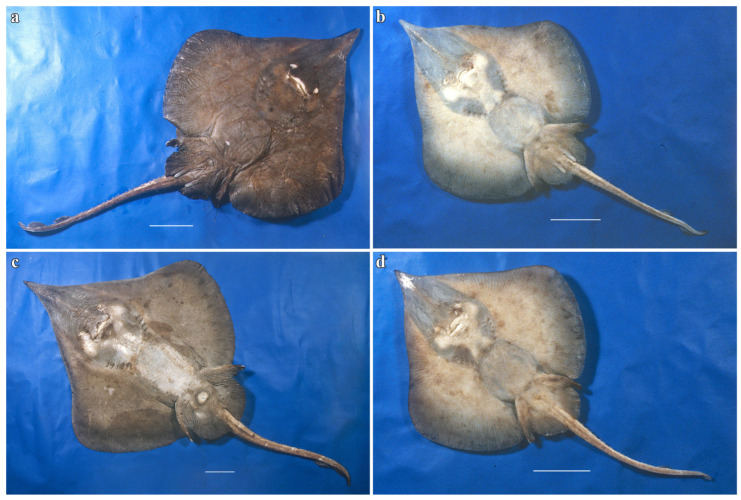
*Leucoraja longirostris* n. sp., variation in ventral coloration shown in photographs of the paratypes ZMH 26261–26264 in total ventral views taken directly after being caught by MFWS. (**a**) ZMH 26261, juvenile male paratype, 477 mm TL; (**b**) ZMH 26262, juvenile male paratype, 381 mm TL; (**c**) ZMH 26263, adult female paratype, 700 mm TL; (**d**) ZMH 26264, juvenile female paratype, 340 mm TL. Scale bars: 5 cm.

**Figure 20:**
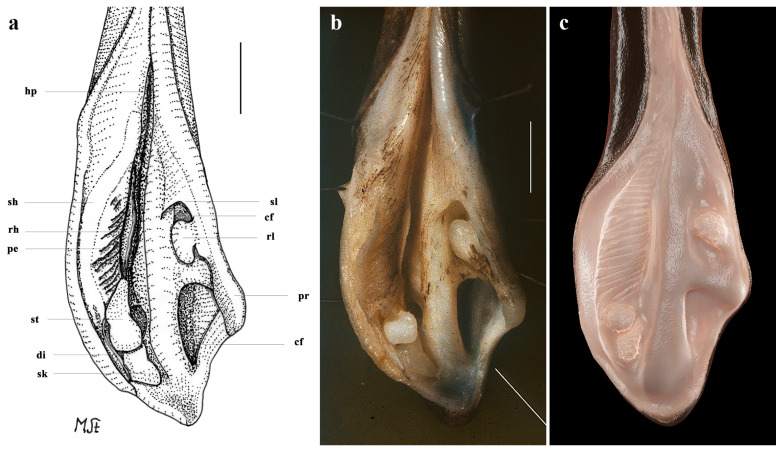
*Leucoraja longirostris* n. sp., ZMH 26260, adult male holotype 711 mm TL, left clasper glans opened. (**a**) Drawing; (**b**) photograph taken directly after being caught by MFWS; (**c**) 3D model. Scale bars: 1 cm. Abbreviations: cf = cleft, di = dike, hp = hypopyle, pe = pent, pr = promontory, rh = rhipidion, rl = roll, sh = shield, sk = spike, sl = slit, st = sentinel.

*External morphology* (Figure 1, Figure 2, Figure 3, Figure 4, Figure 5, Figure 6, Figure 7, Figure 8, Figure 9, Figure 10, Figure 11, Figure 12, Figure 13, Figure 14, Figure 15, Figure 16, Figure 17, Figure 18, Figure 19 and Figure 20). Disc evenly inverse heart-shaped, anterior margins undulated, i.e., concave at anterior sides of snout and at level of nape (disc rhomboid shaped with almost straight to slightly convex anterior margins in paratypes); pectoral apices broadly rounded, posterior pectoral margins evenly convex. Disc width 0.9 (0.9–1.1) times disc length, disc length 2.6 (2.5–2.9) times preorbital snout length and 2.6 (2.4–2.7) times preoral snout length; axis of maximum disc width at 35.6% (31.2–36.8%) TL and 62.5% (58.2–65.1%) of disc length (Figure 1, Figure 2, Figure 3 and Figure 4). Snout very long, tip pointed; snout angle about 65° (69–85°), the narrower angle in larger specimens; preorbital length 21.6% (17.2–22.3%) TL, 6.4 (4.5–6.8) times interorbital width, and 2.5 (2.4–3.1) times in disc width (Figure 5 and Figure 6). Orbits moderately large, diameter 1.1 (1.0–1.2) times interorbital width and 16.7% (16.5–23.6%) of preorbital snout length; spiracle proper aperture length 70.3% (59.4–71.4%) of orbit horizontal diameter; interspiracular space 1.6 (1.6–1.9) times interorbital width; 13 (12–14) pseudobranchial folds in each spiracle (Figure 7). Tail solid, broadly depressed, gradually tapering to tip; length from mid-vent to tip 0.9 (0.8–1.2) times body length from snout tip to mid-vent and 46.8% (47.5–53.6%) TL; ratio tail width to height at level of pelvic tips 1.8 (1.8–1.9) and at level of first dorsal-fin origin 2.0 (1.4–2.2) (Figure 8 and Figure 9). Both dorsal fins of nearly equal size and shape, dorsal fins long but relatively low, their bases confluent (confluent or with short interspace); ratio base length to height of first dorsal fin 2.4 (2.2–4.1) and of second dorsal fin 2.6 (2.4–3.3); postdorsal tail section short, 3.2% (2.5–3.8%) TL and only 0.5 (0.4–0.7) times second dorsal-fin base length; epichordal caudal lobe low, confluent with second dorsal fin (Figure 10). Brownish lateral tail folds along posterior 63.7% (58.1–72.0%) of tail.

Ventral head length 34.5% (29.5–35.1%) TL and 4.9 (4.2–5.4) times internarial width. Preoral snout length 2.9 (2.8–3.6) times mouth width; mouth width 21.8% (18.5–22.6%) of ventral head length and subequal to internarial space; distance between fifth gill slits 58.1% (55.7–67.3%) of distance between first gill slits, the latter 1.7 (1.6–1.9) times internarial space (Figure 11 and Figure 12). Rear margin of nasal flaps fringed; outer edges of nasal curtain with a distinct lobelet, apices rounded, their outer margin smooth; rear margin of curtain weakly fringed by broad and fleshy fringes; isthmus deeply arc-shaped (Figure 13 and Figure 14). Jaws distinctly angled (nearly straight to moderately angled in paratypes); 43 (39–50) close-set parallel tooth rows in upper jaw; individual tooth with low, subcircular base and elongated, conically pointed cusp (Figure 13). The cusps are short and stout in all female and juvenile male paratypes (Figure 14) and intermediately elongated in early subadult male specimen MTUF 30754.

Pelvic-fin outer margin deeply notched, separating short thumb-like anterior lobe with bluntly rounded tip from elongated posterior lobe with broadly angled outer margin and pointed tip; length of posterior lobe 1.4 (1.2–1.5) times length of anterior lobe (Figure 1, Figure 2, Figure 3 and Figure 4). Clasper postcloacal length 45.6% of tail length in adult male holotype and 14.6–16.7% in juvenile male paratypes (Figure 15).

*Squamation* (Figure 1, Figure 3, Figure 5, Figure 6, Figure 7, Figure 8, Figure 9, Figure 10, Figure 15 and Figure 16): dorsally, largely smooth. Wedge-shaped fields of sharp malar thornlets at anterior disc margins level with orbits, integrated into a narrow band of coarse spinules from propterygia onto center of pectoral apex, where it becomes much wider. The rest of the disc, trunk, integument covering the eyeballs dorsally, pelvic fins, claspers and tail are smooth except for scattered spinules on the sides of the tail and prickly dorsal fins and caudal fin. In all paratypes, dorsal side also largely smooth except for prickly dorsal and caudal fins, as well as the following additional squamations: broad band of loosely set sharp spinules from propterygia along anterior disc margins to outer corners of disc (juvenile male paratypes ZMH 26261 and ZMH 26262, juvenile female paratype ZMH 26265, subadult male paratype MTUF 30754); few sharp spinules on rostral tip and a broad band of coarse and more or less densely set spinules from propterygia onto half of outer corners along anterior disc margins (adult female paratype ZMH 26263); broadly triangular area of dense, coarse prickles from propterygia along anterior disc margins to outer corners, widening rearwards, as well as few prickles interorbitally and along back of body (juvenile female paratype ZMH 26264); broad band of loosely set coarse spinules from propterygia along anterior disc margins to outer corners of disc, as well as several spinules on interorbital region, and sparse spinules on pectoral centers and middorsal area from scapular region to origin of first dorsal fin (juvenile female paratype ZMMU P-17803).

Ventrally, largely smooth, only entire snout to level of nostrils densely set with coarse spinules, continued as a narrow band along anterior disc margins to beginning of pectoral apices. In all paratypes, ventral side also largely smooth except for: coarse erect spinules loosely scattered on tip and along sides of snout, continued as a narrow stripe of fine and densely set spinules along anterior half of disc margins to level of mouth (juvenile male paratype ZMH 26261, juvenile female paratype ZMH 26265, subadult male paratype MTUF 30754); spinules only on tip and sides of snout (juvenile male paratype ZMH 26262, juvenile female paratype ZMH 26264); coarse, sharp spinules on snout tip and narrow stripe along anterior disc edge to level of lower jaw but rostral sides almost smooth to level of nostrils, a separate patch of spinules in front of each nostril (adult female paratype ZMH 26263); only one hooked spinule on tip of snout a few ones anterior to tip of pectoral radials (juvenile female paratype ZMMU P-17803).

Thorn counts of the holotype and all paratypes are given in Table 2. Rostral thorns distinct and arranged in an irregular median row along anterior two-thirds of rostrum splitting into two rows from tip of anterior fontanelle rearwards; rearmost thorn almost level with anterior edge of orbits. Supraorbital thorns absent in adult male holotype ZMH 26260 (likewise in all paratypes except for juvenile male ZMH 26261). Preorbital thorn count equal in the holotype and most paratypes but paratype ZMH 26261 with an additional outer thorn on left and right side, and paratype ZMMU P-17803 with an additional one on the left side only. The inner preorbital thorn is much larger than the outer one(s) in all specimens. Apparently, thorn counts in orbito-spiracular and nape-shoulder regions do neither increase nor decrease with growth. However, the number of rostral thorns is reduced in the adult female paratype ZMH 26263 with only about 5 thorns in second last quarter of rostral length plus 3 at anterior end of anterior fontanelle. In the holotype and all paratypes, the median row of thorns originates behind the shoulder and ends before the origin of the first dorsal fin, with the thorns decreasing in size rearwards. In the holotype, an irregular double row of parallel thorns originates directly behind the shoulder girdle on each side of the median row, merging to a single row of parallel thorns at tail origin and dividing into a double row again towards the tip of tail by intermingling with the lateral tail thorns. The parallel thorns reach to the first dorsal-fin origin, the lateral thorns to the second dorsal-fin origin. In paratype ZMH 26261, there is a single parallel row of thorns on each side of the median row, which originates somewhat behind the shoulder girdle and, like in the holotype, reaches to the first dorsal-fin origin. On the tail, there is an additional row of lateral thorns, which intermingles with the row of parallel thorns and, like in the holotype, reaches to the second dorsal-fin origin. In paratype ZMH 26262, there is an irregular parallel row of about 20 ribbed thornlets from pelvic girdle to the first dorsal fin, as well as a lateral row of 20–25 thornlets along the tail to about second dorsal-fin origin. In the holotype and all paratypes, the thorns in the median row are distinct, closely set along tail and subtriangular-based of low conical shape with short, slightly recurving tip. The thorns in the parallel rows have an oval base and are much shorter and less strongly recurved than the median thorns. The bases of all thorns are distinctly and finely ribbed.

**Table 2:** *Leucoraja longirostris* n. sp., thorn counts for all eight type specimens (HT = holotype, PT = paratype).

	HT ZMH 26260	PT ZMH 26261	PT ZMH 26262	PT ZMH 26263	PT ZMH 26264	PT ZMH 26265	PT MTUF 30754	PT ZMMU P-17803
rostral thorns	~15	~20	~15	~8	~10	5	~10	~14
preorbital thorns	2/2	3/3	2/2	2/2	2/2	2/2	2/2	3/2
supraorbital thorns	-	1/1	-	-	-	-	-	-
postorbital thorns	1/1	1/1	2/1	1/1	1/2	1/1	2/2	1/1
supraspiracular thorns	1/1	1/1	1/0 (right one lost?)	1/1	1/1	1/1	1/1	1/1
interspiracular thorns	1/1	1/1	1/1	1/1	1/1	1/1	1/1	0/1 (left one lost?)
median nuchal thorns	4 (5th lost?)	3	4	3	5	3	4	3
lateral nuchal thorns, left/right	1/1	1/1	1/0	3/2	0	0	1/1	0
suprascapular thorns	1	2	2	2	1	2	1	2
lateral scapular thorns, left/right	1/1	0/1	2/2	2/2	1/1	1/1	1/1	0/1
scapular thorns, left/right	3/3	2/2	2/2	3/3	3/3	2/2	2/2	3/3
median thorns on body	9	7	7	7	7	9	8	9
median thorns on tail	28	27	27	30	30	32	30	29
interdorsal thorns	0	0	0	0	0	0	0	0
parallel thorns on body, left/right	15/15	6/6	1/1	~13/~13	0/1	0/1	2/2	0/0
parallel thorns on tail, left/right	~30/~30	~25/~30	~20/~20	~35/~35	~25/~25	~20/~20	~25/~20	~20/~20
lateral thorns on tail, left/right	~35/~35	~20/~20	~20/~25	~40/~40	~25/~25	~15/~15	~20/~20	~15/~15
alar thorns	2 rows of 20 each	-	-	-	-	-	-	-

Adult male holotype ZMH 26260 with a very elongated field of 2 longitudinal and 20 transverse rows of sharp, claw-like alar thorns across the inner apex; the alar thorns are erectable from individual dermal pockets (Figure 16).

*Coloration*: when fresh (holotype ZMH 26260 and paratypes ZMH 26261–26265; Figure 17, Figure 18 and Figure 19): dorsal side (Figure 17) plain medium-brown, with grayish mucus coverage. Tips of anterior and outer margin of posterior pelvic lobes blackish-brown; eyes dusky-bluish. Clasper stem grayish-brown, glans dark brown. Ventral side (Figure 18) plain grayish on disc and pelvic fins, snout dusky grayish, sensory pores not marked dark. Tips of anterior and margin of posterior pelvic lobes chestnut-brown. Anterior distal edge of anterior lobe creamy white. Claspers and underside of tail brown but tail with pale edging in anterior half. Nasal flaps and jaws sharply contrasting white, jaw edges pale only. The dorsal coloration is largely plain medium-brown or grayish brown also in the paratypes but the ventral coloration is highly variable (Figure 19). In juvenile male paratype ZMH 26261, dorsal side as in holotype ZMH 26260. Ventral side (Figure 19a) plain dark brown with dark grayish mucus layer, tail somewhat lighter brownish. Disc margins with blackish shade as tips and margins of pelvic lobes. Anterior distal edge of anterior pelvic lobe narrowly creamy. Tip of clasper light grayish, lateral tail folds blackish brown. Nasal flaps, jaws, mouth angles, and fringes of nasal curtain strongly marked white, cloaca pale. In juvenile male paratype ZMH 26262, dorsal side medium grayish-brown, rostral sides creamy semi-transparent; eyes dusky-bluish. Tips of anterior pelvic lobes dark brown, dorsal fins and caudal fin grayish, tail folds pale. Ventral side (Figure 19b) pale grayish-white, pectoral-fin centers darker towards margins, snout also slightly darker. Few irregular speckles of dark brown from snout along disc margins. Cloacal region pale. Anterior pelvic lobes dusky in distal two-thirds and broad darker grayish margin of posterior lobes. Tail irregularly and vaguely spotted brown on gray, lateral folds pale. Nasal flaps, fringes of nasal curtain and jaws creamy. Pale creamy marking of wineglass-shape from outer jaws to shoulder girdle, “standing” on the latter. In adult female paratype ZMH 26263, dorsal side medium grayish-brown, darker to disc margins, and covered with grayish mucus layer. Brown shade more intense along back of body and on pectoral centers, also along entire tail; eyes dusky-bluish. Anterior pelvic lobes blackish-brown towards the tip, posterior lobes darker to margins. Dorsal fins and caudal fin brownish. Ventral side (Figure 19c) also camouflaged by firm layer of lead-grayish mucus, midline of body, belly, cloacal region, and center of pectoral fins in fact pale whitish, tending to darken outwards. Snout and internasal space dark-brown, as well as margin around disc, and posterior pelvic lobes gradually so from inner parts towards margins. Broad triangle at lower jaw dark brown. Anterior pelvic lobes dark brown towards tip with light marking at anterior distal edge. Nasal flaps, lobelet at outer margin of nasal curtain and jaws white. Central light area reaching forward to sides of nasal region. Tail wish-washy medium brown, tail folds dark. In juvenile female paratype ZMH 26264, dorsal side plain medium grayish-brown, slightly darker than ventrally; eyes dusky-bluish. Tips of anterior pelvic lobes dusky; pelvic fins, tail, dorsal and caudal fins as disc. Ventral side (Figure 19d) similar as in paratype ZMH 26262 but generally darker, with ground color pale grayish-brown, pectoral-fin centers darker towards margins, snout also darker. Irregular speckles of dark brown from snout along disc margins. Cloacal region pale. Anterior pelvic lobes dusky in distal two-thirds and broad darker grayish margin of posterior lobes. Tail irregularly and vaguely spotted brown on gray, lateral folds pale. Nasal flaps, fringes of nasal curtain and jaws creamy. Pale creamy marking of wineglass-shape from outer jaws to shoulder girdle, “standing” on the latter. In paratype ZMH 26265, dorsal side also medium-grayish brown and camouflaged by grayish mucus coverage. Brown shade most intense along midline of head, body and along tail, as well as along disc margins. Dorsal fins and upper caudal fold grayish-brown. Lateral tail folds dark at beginning, pale rearwards. Ventral side also camouflaged by grayish mucus coverage; similar as in paratype ZMH 26262, pale gray, becoming darker towards brown shade on disc and posterior pelvic margin. Anterior pelvic lobes blackish towards tip, with white marking at distal anterior margins. Underside of tail brownish. Nasal flaps, fringes of nasal curtain and jaws white. Interbranchial space to lower jaw and sides of jaw angles pale grayish and shaped like a wineglass standing on shoulder girdle. Anterior belly, gill regions and their anterior extensions to level of nostrils bluish-black and thus marking off the lighter “wineglass”.

Color in preservative (all type specimens; Figure 1, Figure 2, Figure 3 and Figure 4): coloration of holotype and ZMH paratypes as described above with all markings still apparent but grayish parts rather brownish and whitish parts rather beige. Subadult male paratype MTUF 30754 is plain grayish-brown on both surfaces, somewhat darker below; tips of anterior pelvic lobes and margins of posterior lobes dark brown; thorns light yellowish. Ventral side with nasal flaps, fringes of nasal curtain, jaws, and anterodistal edge of anterior pelvic lobes marked pale whitish. Remains of a coffee-brown mucus layer centrally on underside of disc, but mucus pores not marked black. Juvenile female paratype ZMMU P-17803 creamy brown on both surfaces, somewhat lighter below; thorns light yellowish, eyes bluish. Side areas of rostrum more distinctly semi-transparent and lighter than in paratype MTUF 30754.

*Clasper external morphology* (Figure 20):

The adult male holotype claspers are robust and relatively long, with their tips reaching to about three fifths of tail length. The glans is only little widened and rather short, less than one third of the clasper length. Proximal half of the clasper stem is dorsally medium grayish-brown as the ground color of disc and pelvic fins, the distal half with the glans dark brown; ventrally, the claspers are totally medium grayish-brown as the disc, pelvic fins and underside of tail. Outer surfaces of both, dorsal and ventral glans lobes show no external components.

The opened glans of the left clasper (Figure 20) displays on ventral lobe the components: shield (sh) along proximal four fifths of the glans as outer edge but curving toward median axis in its proximal two fifths; the shield is entirely covered by integument, without a cutting, free cartilaginous outer edge. The pent (pe) of pleated integument covers the inner area of the shield’s median two fourths and downward into the clasper groove; the pent is medium brown colored and thus marked of from the predominantly white inner glans surfaces. The rhipidion (rh) is a thin, narrow fold running within the clasper groove parallel to the proximal two thirds of the pent, with a porous inner surface; its color is medium brown like the pent. The distal part of the shield is a vertical cartilaginous wall covered with thin integument and forms the component dike (di) along most of the combined length of sentinel and spike. Components on inner dorsal lobe: most apparent are two deep clefts (cf) between the axial and dorsal cartilages; the distal cleft appears longer than the proximal cleft, because the upper end of the latter is hidden under component slit (sl) spanning across from the axial cartilage to the outer margin of the dorsal lobe. A seeming terminal bridge between both clefts is not supported by cartilage but formed by ligament only. Component roll (rl) is placed over the distal part of the proximal cleft as a bulbous integumental element, which in preserved condition appears somewhat collapsed and less apparent; the roll is attached to the proximal part of component promontory (pr) forming the major part of outer margin of the dorsal lobe; the promontory is covered by thin integument, and its distal tip is not free and sticking out but connected with the distal outer margin of the lobe. This differs from the appearance of the promontory in congeners (cp. Stehmann [7], see Discussion). Both the latter components, roll and promontory, are diagnostic for the genus *Leucoraja* and best detectable in fresh specimens (see Figure 20b). Both further ventral lobe components sentinel (st) and spike (sk) are placed over the distal end of the clasper groove, with the spike underneath the sentinel and the more distal element; both are cartilaginous tips covered by fleshy integument.

**Figure 21:**
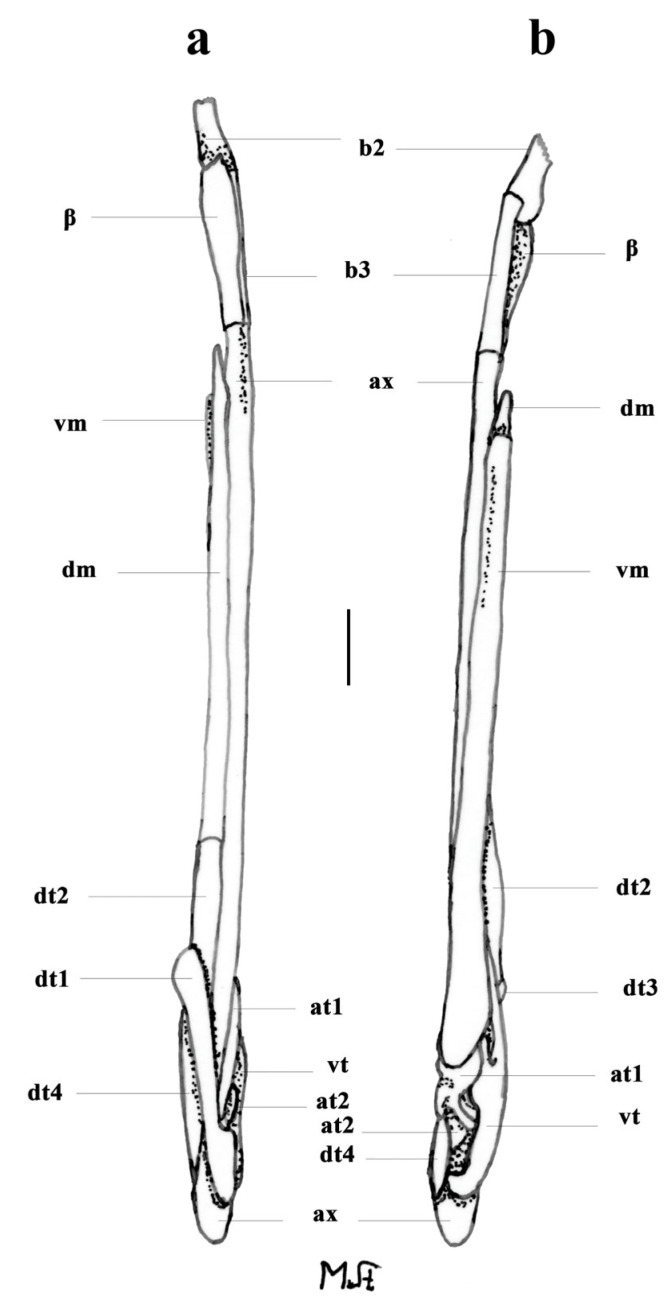
*Leucoraja longirostris* n. sp., ZMH 26260, adult male holotype, 711 mm TL, entire left clasper skeleton drawn in (**a**) dorsal and (**b**) ventral views. Scale bar: 1 cm. Abbreviations: at1 and at2 = accessory terminal cartilages 1 and 2, ax = axial cartilage, β = beta cartilage, b2 and b3 = clasper stem basal elements 2 and 3, dm = dorsal marginal cartilage, dt1 to dt4 = dorsal terminal cartilages 1 to 4, vm = ventral marginal cartilage, vt = ventral terminal cartilage.

**Figure 22:**
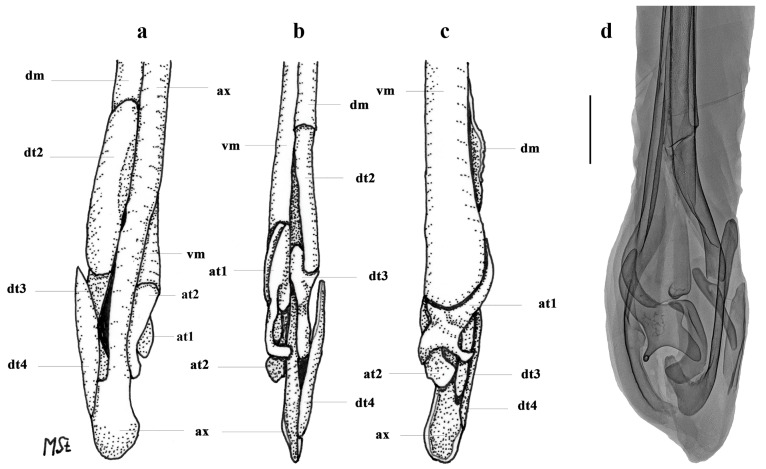
*Leucoraja longirostris* n. sp., ZMH 26260, adult male holotype, 711 mm TL, terminal clasper skeleton. (**a**–**c**) Drawings of dissected left skeleton in (**a**) dorsal, (**b**) frontal, and (**c**) ventral views, with dorsal terminal 1 (dt1) and ventral terminal (vt) cartilages removed; (**d**) radiograph of right skeleton in opened view (image reversed). Scale bar: 1 cm. Abbreviations: at1 and at2 = accessory terminal cartilages 1 and 2, ax = axial cartilage, dm = dorsal marginal cartilage, dt2 to dt4 = dorsal terminal cartilages 2 to 4, vm = ventral marginal cartilage.

**Figure 23:**
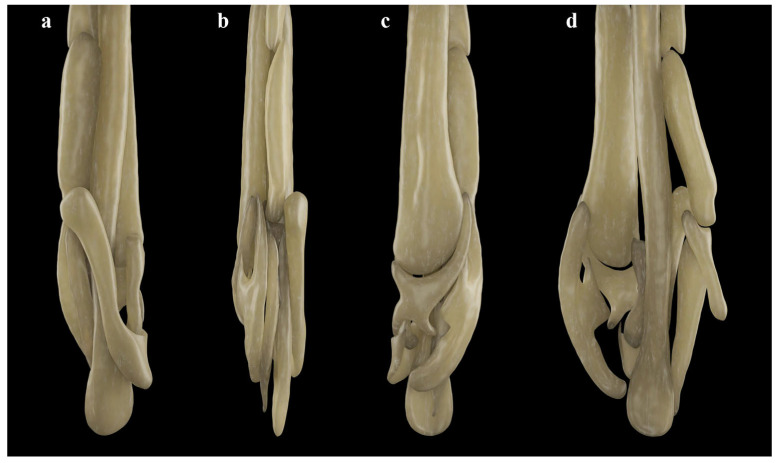
*Leucoraja longirostris* n. sp., ZMH 26260, adult male holotype, 711 mm TL, 3D models of complete left terminal clasper skeleton in (**a**) dorsal, (**b**) frontal, (**c**) ventral, and (**d**) opened views.

**Figure 24:**
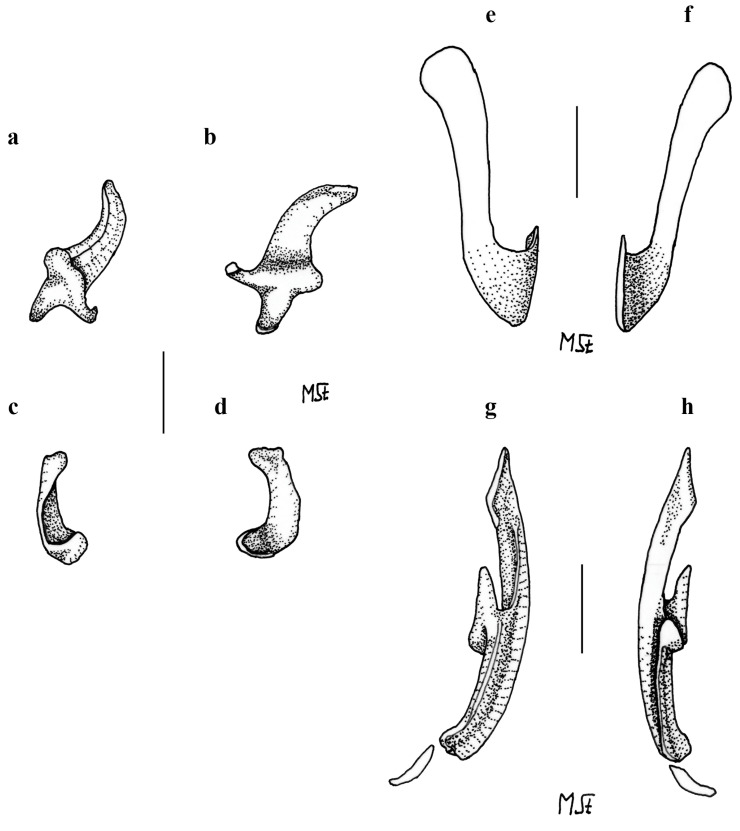
*Leucoraja longirostris* n. sp., ZMH 26260, adult male holotype, 711 mm TL, drawings of isolated cartilages from left terminal clasper skeleton. (**a**,**b**) Accessory terminal 1 cartilage (at1) in (**a**) ventral and (**b**) dorsal views; (**c**,**d**) accessory terminal 2 cartilage (at2) in (**c**) ventral and (**d**) dorsal views; (**e**,**f**) dorsal terminal 1 cartilage (dt1) in (**e**) dorsal and (**f**) ventral views; (**g**,**h**) ventral terminal cartilage (vt) plus unnamed fibrocartilage in (**g**) ventral and (**h**) dorsal views. Scale bars: 1 cm.

**Figure 25:**
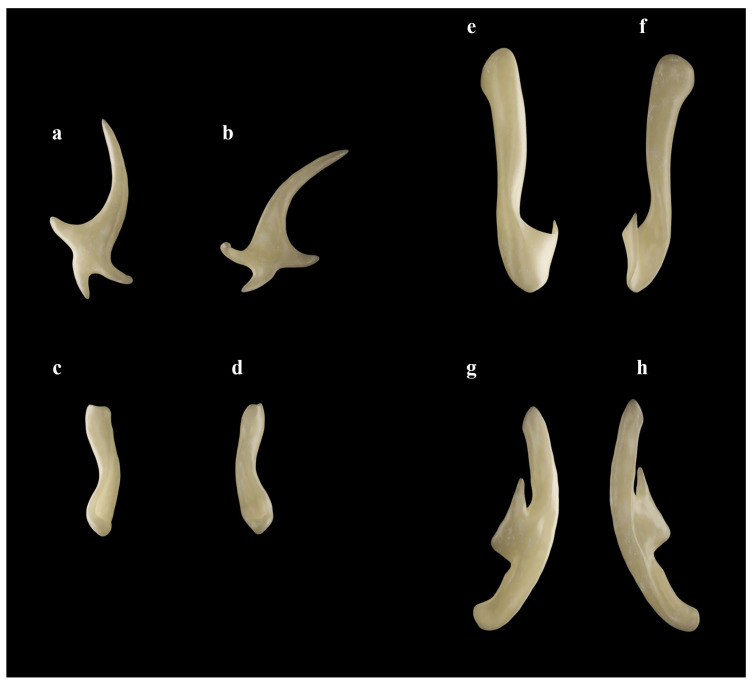
*Leucoraja longirostris* n. sp., ZMH 26260, adult male holotype, 711 mm TL, 3D models of isolated cartilages from left terminal clasper skeleton. (**a**,**b**) Accessory terminal 1 cartilage (at1) in (**a**) ventral and (**b**) dorsal views; (**c**,**d**) accessory terminal 2 cartilage (at2) in (**c**) ventral and (**d**) dorsal views; (**e**,**f**) dorsal terminal 1 cartilage (dt1) in (**e**) dorsal and (**f**) ventral views; (**g**,**h**) ventral terminal cartilage (vt) in (**g**) ventral and (**h**) dorsal views.

**Figure 26:**
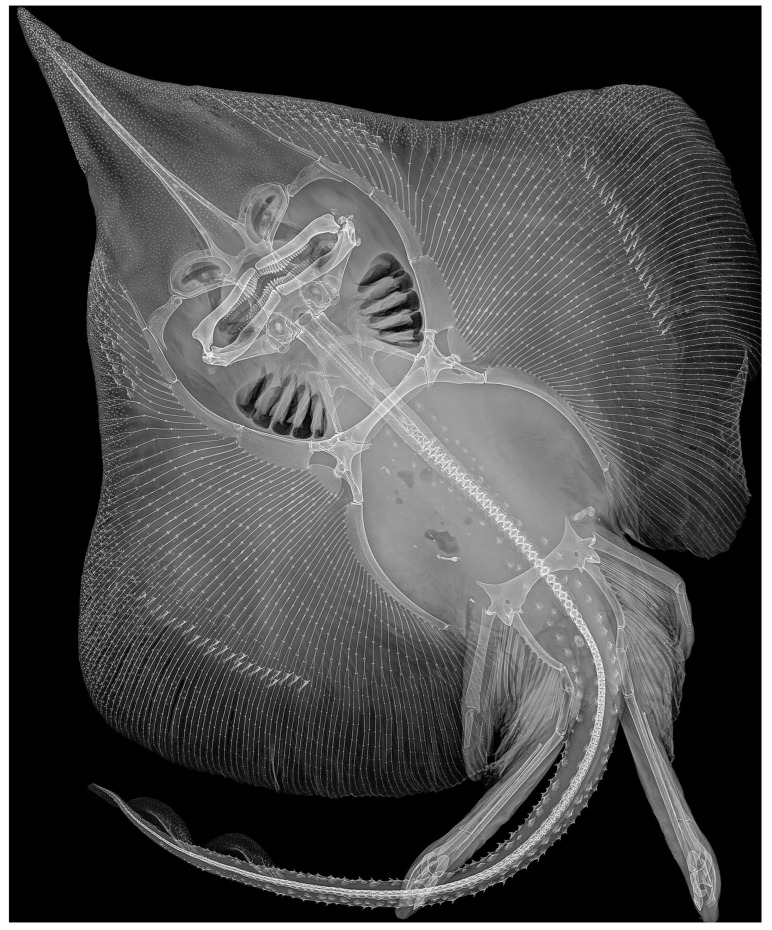
*Leucoraja longirostris* n. sp., ZMH 26260, adult male holotype, 711 mm TL, radiograph in total dorsal view.

*Clasper skeleton* (Figure 21, Figure 22, Figure 23, Figure 24, Figure 25 and Figure 26):

The total dorsal and ventral views of the entire clasper skeleton (Figure 21) are only a simplified overview to illustrate the skeleton’s composition and the positions of the dorsal terminal 1 and ventral terminal cartilages, which are both removed in Figure 22a–c but drawn separately in close-up views. The drawings of the terminal clasper skeleton (Figure 22a–c) are complemented by a radiograph of the opened clasper glans (Figure 22d) and these images are contrasted by 3D models of the complete left terminal clasper skeleton (Figure 23). Correspondingly, the drawings of isolated terminal clasper cartilages (Figure 24) are contrasted by 3D models of the same isolated cartilages (Figure 25). Three basal cartilages (b1 to b3) connect the pelvic girdle with the head of the axial cartilage (ax): see Figure 21 and Figure 26. The beta-cartilage (β) is elongated spatulate, attached jointly with the b3-cartilage to the head of the axial, and its proximal tip is extended to over the distal end of the b2-cartilage. The clasper stem consists of the axial, dorsal marginal (dm), and ventral marginal (vm) cartilages, forming the major part of the clasper groove; the axial runs along the clasper skeleton, with a bluntly rounded tip; proximally, the bluntly pointed end of the dm is longer than the rectangular tip of the vm; but in total, the dm is much shorter than the vm. The dm is almost evenly narrowly plate-like, with a horizontal distal edge and a thickened, undulated outer distal margin appearing in the ventral view of the terminal skeleton (Figure 22c). In contrast, the vm widens in its distal third to become spoon-shaped with a rounded distal contour and curves distally around the ax to appear in the dorsal view of the terminal skeleton (Figure 22a). The terminal glans skeleton consists of eight cartilages, i.e., the dorsal terminals dt1 to dt4, the ventral terminal (vt), and the accessory terminal cartilages at1 and at2, plus a tiny separate spindle-shaped fibrocartilage (*sensu* McEachran and Miyake [39] and Leible and Stehmann [40]) between the distal tips of the dt1 and the vt. The dt1 (Figure 24e,f and Figure 25e,f) is narrowly strap-shaped and runs slightly diagonally over the terminal skeleton from its proximal end, over the dt2 to its widened distal third, curving around the axial onto the ventral side. The dt2 is very elongated, plate-like, and attached to the entire distal edge of the dm. The dt3 is club-shaped, with the wider proximal part attached underneath the distal end of the dt2, and the dt3, almost entirely hidden under the somewhat concave, elongated spindle-shaped dt4 connecting distally with the tip of the ax; the tip of the dt3 externally forms the component promontory. The ventral terminal (vt) (Figure 24g,h and Figure 25g,h) is weakly arc- rather than J-shaped, attached proximally underneath the outer distal margin of the vm, medially attached with a short finger-like process to a notch on the proximal long, outer branch of the at1. Finally, it is distally linked by a tough ligament bridge to the distal edge of the dt1, curving onto the ventral side; the proximal tip of the vt is triangularly pointed, the lateral lamella very narrow, and in the dorsal view, distally from the median process, the latter continues as a thin vertical wall (forming the external component dike) alongside a deep groove. The spindle-shaped, tiny separate fibrocartilage, drawn with the vt, remains unnamed. It was found within the ligament bridge between distal tips of the vt and dt1. Such calcifications within ligaments are occasionally found (cp. Backman [41], p. 37; Stehmann [7], p. 100 and Plate 8 dt4; McEachran and Miyake [39], pp. 286, 288; Leible and Stehmann [40], p. 182) and are not part of the principal clasper skeleton. The at1, with its four tips (Figure 22d, Figure 24a,b and Figure 25a,b), is extraordinary. It is attached to the vm around most of the rounded distal vm-edge; the curved outer distal tip externally supports the component sentinel. The at2 (Figure 24c,d and Figure 25c,d) has the shape of a baseball glove, is weakly curved, and is attached to the very inner end of the distal vm edge alongside the at1, and is located underneath the at1; its bluntly rounded, concave distal tip supports the external component spike.

**Figure 27:**
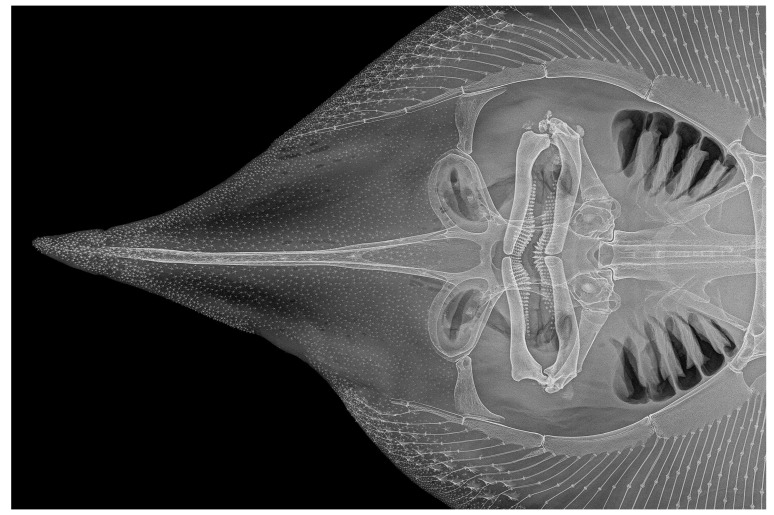
*Leucoraja longirostris* n. sp., ZMH 26260, adult male holotype, 711 mm TL, radiograph of cranium and snout in dorsal view.

**Figure 28:**
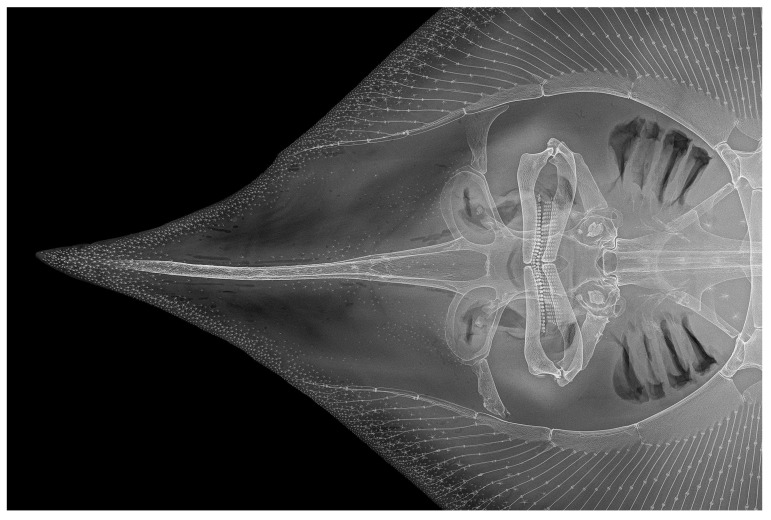
*Leucoraja longirostris* n. sp., ZMH 26263, adult female paratype, 700 mm TL, radiograph of cranium and snout in dorsal view.

**Figure 29:**
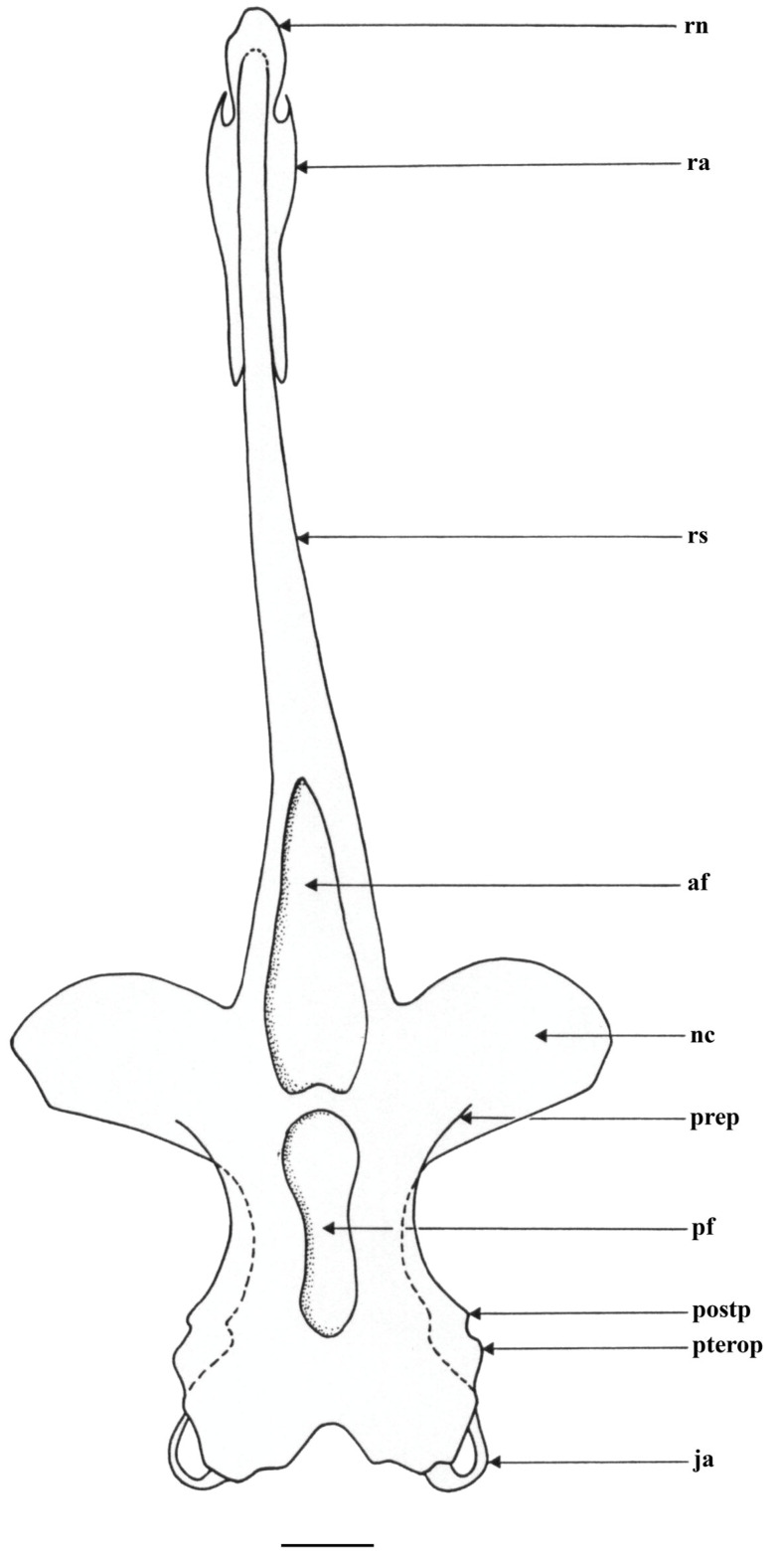
*Leucoraja longirostris* n. sp., MTUF 30754, early subadult male paratype, 592 mm TL, drawing of cranium and snout in dorsal view semi-schematically reconstructed in combination of radiographs and dissected snout. Scale bar: 1 cm. Abbreviations: af = anterior fontanelle, ja = jugal arch, nc = nasal capsule, pf = posterior fontanelle, postp = postorbital process, prep = preorbital process, pterop = pterotic process, ra = rostral appendix, rn = rostral node, rs = rostral shaft.

*Cranium* (Figure 27, Figure 28 and Figure 29): the cranium shown by radiographs of adult male holotype ZMH 26260 (Figure 27) and adult female paratype ZMH 26263 (Figure 28), as well as drawing of partially dissected cranium of early subadult male paratype MTUF 30754 (Figure 29); Table 3 provides cranial morphometrics measured from radiographs.

**Table 3:** *Leucoraja longirostris* n. sp., morphometrics of the cranium. Individual values for the adult male holotype (ZMH 26260), adult female paratype (ZMH 26263), and early subadult male paratype (MTUF 30754), ranges for all juvenile paratypes (*n* = 5), as well as means and standard deviations (SD) for all eight type specimens. Values of the smallest juvenile female paratype (ZMMU P-17803) only partially included due to distortion of the rostral shaft. Proportional values are expressed as percentages of nasobasal length (NBL) except for the minimum, maximum, and mean of the NBL in millimeters.

	Holotype, ZMH 26260, Adult Male, 711 mm TL		Paratype, ZMH 26263, Adult Female, 700 mm TL		Paratype, MTUF 30754, Early Subadult Male, 592 mm TL		Minimum (*n* = 5)	Maximum (*n* = 5)	Mean (*n* = 8)	SD
	mm	% NBL	mm	% NBL	mm	% NBL	% NBL	% NBL	% NBL	
cranium TL	201.9	348.8	208.1	357.4	183.8	358.1	317.8	337.2	338.5	16.8
nasobasal length (NBL)	57.9	100.0	58.2	100.0	51.3	100.0	23.9	36.9	40.4	
max. ethmoidal width	73.4	126.8	71.4	122.6	66.7	130.0	127.8	130.4	128.1	2.5
min. dorsal interorb. width	24.0	41.5	23.8	40.9	20.0	39.0	40.1	43.9	41.1	1.4
min. internasal width	15.5	26.8	15.0	25.8	14.1	27.5	26.6	29.9	27.4	1.2
max. width nasal apertures	27.5	47.6	25.4	43.6	25.6	49.8	44.7	46.7	46.5	1.9
min. ventral interorb./basal plate width	19.2	33.2	18.6	31.9	17.2	33.5	32.1	35.5	33.3	1.4
max. width otic region	38.3	66.1	39.0	66.9	34.9	68.1	68.0	71.4	68.9	2.0
max. width jugular	40.2	69.5	39.7	68.1	36.2	70.6	71.9	74.6	71.5	2.0
rostral shaft length	143.7	248.3	149.6	256.9	132.3	257.8	217.8	237.2	238.2	16.7
rostrum base width	18.1	31.2	18.8	32.4	18.4	35.8	32.0	38.1	34.6	2.6
postnasal length orbit region	19.6	33.8	19.2	33.0	15.6	30.4	28.7	33.3	31.3	2.1
length otic region	17.9	31.0	18.6	31.9	17.0	33.2	32.2	36.8	33.7	2.1
postoccipital length jugal arches	2.1	3.7	2.0	3.4	1.8	3.5	1.3	3.0	2.6	0.9
tip rostrum to tip ant. fontanelle	114.6	198.0	119.7	205.6	105.9	206.4	171.1	185.9	188.3	15.1
tip to end ant. fontanelle	155.2	268.0	160.4	275.4	139.8	272.5	231.5	251.8	253.9	18.2
tip to tip post. fontanelle	159.0	274.6	163.6	281.0	142.0	276.7	236.5	259.3	259.5	18.5
tip to end post. fontanelle	183.5	317.1	189.8	326.0	167.9	327.2	283.8	304.5	305.7	18.3
tip to level ant. propterygia	86.4	149.3	90.7	155.7	78.5	153.0	112.2	132.7	134.8	18.1
tip to level max. ethmoidal width	146.3	252.7	155.0	266.2	135.9	264.9	223.1	242.2	244.9	17.0
tip to symphysis upper jaw	163.1	281.7	174.0	298.8	148.7	289.8	250.8	263.3	270.7	19.2
ant. fontanelle length	40.5	70.0	40.8	70.1	33.8	65.8	59.9	66.8	65.7	3.7
ant. fontanelle max. width	12.1	21.0	11.3	19.5	12.3	24.0	18.9	25.9	22.5	2.6
distance betw. ant. + post. fontanelles	3.9	6.7	3.4	5.8	2.1	4.2	4.1	7.1	5.5	1.2
post. fontanelle length	24.6	42.4	26.4	45.3	26.1	50.8	45.7	47.2	46.4	2.3
post. fontanelle min. width	8.0	13.9	6.2	10.7	5.4	10.5	8.6	12.5	10.9	1.7
post. fontanelle max. width anteriorly	10.2	17.6	10.3	17.8	8.5	16.6	13.1	17.8	16.2	1.8
post. fontanelle max. width posteriorly	8.0	13.9	6.9	11.9	6.2	12.1	11.7	15.5	12.8	1.4
angle post. edge nasal capsules, °	66.0		70.0		72.0		72.0	80.0	73.1	4.3

Rostral cartilage very long, length of rostral shaft 71.2% (68.5–72%) of cranium length and 2.5 (2.2–2.6) times nasobasal length; maximum ethmoidal width 36.4% (34.3–40.6%) of cranium length and 4.1 (3.4–4) times rostral base width; rostral base width 12.6% (12.6–17.5%) of rostral shaft length; minimum dorsal interorbital width 32.7% (30–33.6%) of maximum ethmoidal width; postnasal length of orbit region 33.8% (28.7–33.3%) of nasobasal length; length of otic region 31% (31.9–36.8%) of nasobasal length; maximum width of otic region 52.1% (52.3–55.6%) of maximum ethmoidal width. Both dorsal fontanelles separated by relatively deep cartilaginous bridge. Anterior fontanelle elongated egg-shaped, with bluntly pointed tip and nearly straight transverse posterior edge, its length 20.1% (18.4–19.8%) of cranium length. Posterior fontanelle gourd-shaped, with weak constriction at mid-length; the broader anterior part subquadrate, with straight to weakly convex transverse edge, the posterior part bluntly pointed; length of posterior fontanelle 12.2% (12.7–14.9%) of cranium length. Judging from the partially dissected cranium of paratype MTUF 30754 (Figure 29), the rostral appendices are long, their length about 80% of nasobasal length and about 2.6 times in rostral shaft length; anterior part of rostral appendices a thin cartilaginous plate with an oval notch at its outer margin, posterior part consists of a finger-like process linked to the rostral shaft by connective tissue.

**Figure 30:**
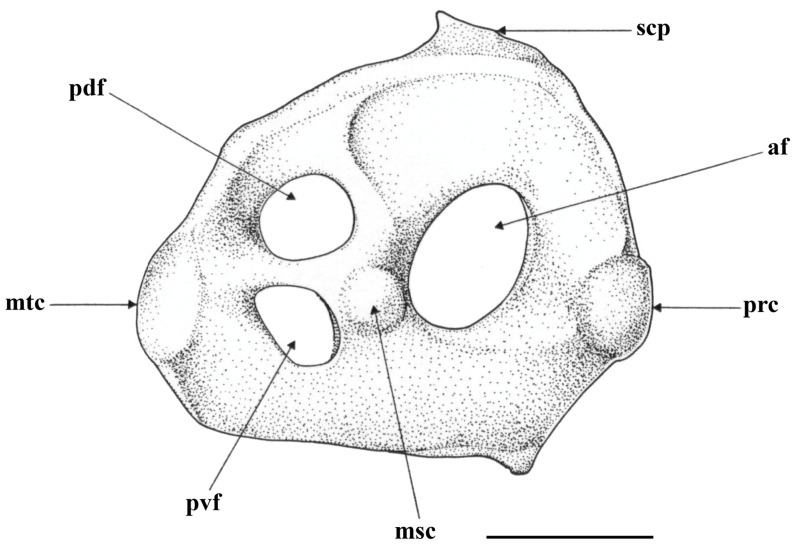
*Leucoraja longirostris* n. sp., MTUF 30754, early subadult male paratype, 592 mm TL, drawing of dissected right scapulocoracoid. Scale bar: 1 cm. Abbreviations: af = anterior fenestra, msc = mesocondyle, mtc = metacondyle, pdf = postdorsal fenestra, prc = procondyle, pvf = postventral fenestrae, scp = scapular process.

*Scapulocoracoid* (Figure 30): element shown by drawing of the dissected right scapulocoracoid of early subadult male paratype MTUF 30754 (Figure 30); measurements are presented in Table 4.

**Table 4:** *Leucoraja longirostris* n. sp., morphometrics of the right scapulocoracoid based on the dissected element of the early subadult male paratype MTUF 30754. Proportional values are expressed as percentages of the element’s maximum length.

	mm	% max. Length
maximum length	30.6	100.0
maximum height	26.9	86.8
height at rear corner	18.7	60.3
pre-mesocondyle length	16.4	53.6
post-metacondyle length	14.2	46.4
anterior fenestra height	8.3	27.1
anterior fenestra length	7.0	22.9
height postdorsal fenestra	5.4	17.6
length postdorsal fenestra	6.5	21.2
height postventral fenestra	4.9	16.0
length postventral fenestra	5.1	16.7

Dissected element subquadrangular, only weakly elongate, its length 1.1 times its height; pre-mesocondyle length slightly longer than post-mesocondyle length and 53.6% of maximum length, height at rear corner 69.5% of maximum height; one large anterior fenestra (af) without anterior bridge; single postdorsal fenestra (pdf) somewhat larger than the single postventral fenestra (pvf); postdorsal margin gently sloping, rear corner poorly developed.

**Figure 31:**
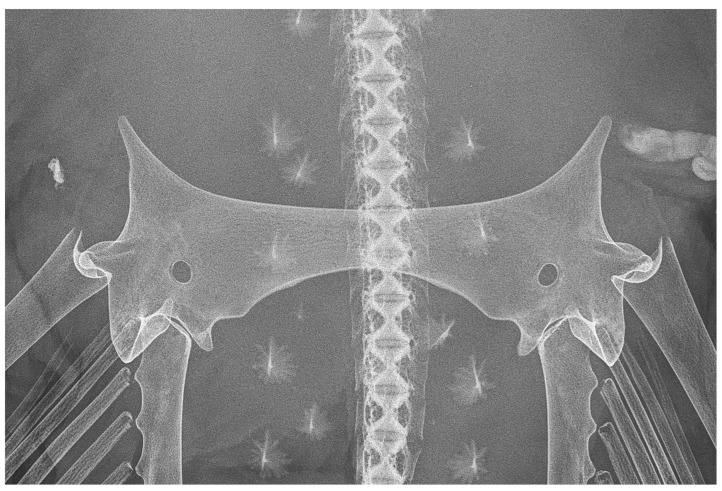
*Leucoraja longirostris* n. sp., ZMH 26260, adult male holotype, 711 mm TL, radiograph of pelvic girdle in dorsal view.

**Figure 32:**
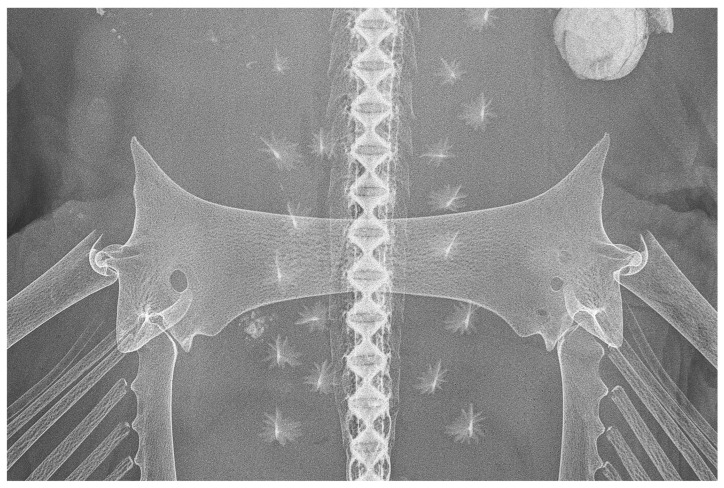
*Leucoraja longirostris* n. sp., ZMH 26263, adult female paratype, 700 mm TL, radiograph of pelvic girdle in dorsal view.

**Figure 33:**
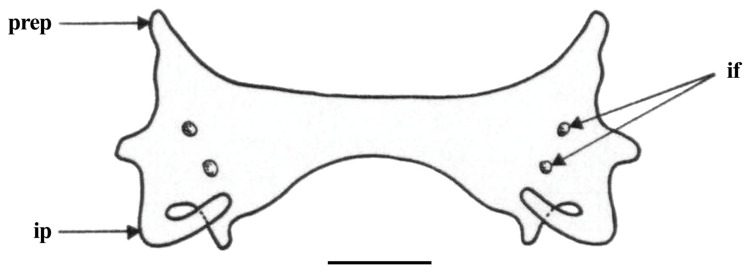
*Leucoraja longirostris* n. sp., MTUF 30754, early subadult male paratype, 592 mm TL, drawing of pelvic girdle in dorsal view, somewhat schematized after radiograph. Scale bar: 1 cm. Abbreviations: if = iliac foramina, ip = iliac process, prep = prepelvic process.

*Pelvic girdle* (Figure 31, Figure 32 and Figure 33): the pelvic girdle shown by radiographs of adult male holotype ZMH 26260 (Figure 31) and adult female paratype ZMH 26263 (Figure 32), as well as drawing of early subadult male paratype MTUF 30754 (Figure 33); Table 5 provides pelvic measurements taken from radiographs.

**Table 5:** *Leucoraja longirostris* n. sp., morphometrics of pelvic girdle based on radiographs, plus interorbital width dorsally and shoulder girdle maximum width as reference values. Individual values for the adult male holotype (ZMH 26260), adult female paratype (ZMH 26263), and early subadult male paratype (MTUF 30754), ranges for all juvenile paratypes (*n* = 5), as well as means and standard deviations (SD) for all eight type specimens. Proportional values are expressed as percentages of pelvic girdle maximum width (PGW) except for the minimum, maximum, and mean of PGW in millimeters.

	Holotype, ZMH 26260, Adult Male, 711 mm TL		Paratype, ZMH 26263, Adult Female, 700 mm TL		Paratype, MTUF 30754, Early Subadult Male, 592 mm TL		Minimum (*n* = 5)	Maximum (*n* = 5)	Mean (*n* = 8)	SD
	mm	% PGW	mm	% PGW	mm	% PGW	% PGW	% PGW	% PGW	
interorbital width dorsally	24.0	39.6	23.8	37.8	20.0	38.6	39.2	45.8	40.8	2.7
shoulder girdle max. width	85.1	140.6	98.5	156.3	-	-	154.1	165.0	155.3	7.4
pelvic girdle max. width (PGW)	60.6	100.0	63.0	100.0	51.8	100.0	22.9	38.0	41.2	
median transverse thickness	5.5	9.2	8.3	13.2	6.1	11.8	11.9	13.6	12.1	1.3
length of prepelvic process (from level PGW)	16.0	26.4	13.9	22.0	13.9	26.8	24.8	28.1	25.9	1.8
length of prepelvic proc. (from level ant. edge pelvic girdle)	10.0	16.5	9.9	15.7	7.9	15.2	13.3	17.8	15.8	1.4
depth of posterior arc (from level PGW)	9.6	15.8	10.9	17.3	8.3	16.0	15.3	17.9	16.4	1.0
depth of post. arc (from level post. edge pelvic girdle)	8.8	14.5	6.3	10.0	8.0	15.4	12.0	15.3	13.8	1.9
iliac foramina number	2		2		2		2	2	2	

Ischiopubic bar massive, with nearly straight anterior contour; iliac regions massive, with two foramina per side; posterior contour deeply concave arc-shaped (somewhat shallower in adult female paratype ZMH 26263). Prepelvic processes short, solid, conical, somewhat inclined outwards, and with bluntly pointed tip, their length from level pelvic girdle maximum width 2.9 (1.7–2.3) times median thickness of ischiopubic bar. Iliac processes moderately long, solid, slenderly finger-like, and oriented anteriorly; transition to iliac regions well marked as blunt elevation. Axis of pelvic girdle maximum width somewhat anterior (clearly anterior in adult female paratype ZMH 26263) to rear contour of ischiopubic bar. Proportionally, the pelvic girdle of the adult female paratype (ZMH 26263) is only slightly wider than that of the other paratypes and the adult male holotype: pelvic girdle maximum width 9% TL in adult female paratype, 8–8.8% TL in the other paratypes and the holotype. In relation to the shoulder girdle maximum width, the pelvic girdle is clearly widest in the adult male holotype: pelvic girdle width 71.1% of shoulder girdle width in the adult male holotype, 60.6–64.9% in the paratypes.

*Skeletal meristics* (from radiographs, Table 1, Figure 26): trunk vertebrae (Vtr): 29 (27–30); predorsal tail vertebrae (Vprd): 62 (60–64); total predorsal vertebrae: 91 (88–94), terminal vertebrae (Vterm): 45 (36–~47); total vertebrae (Vtotal): 136 (127–~136); pectoral radials, left: 70 (69–72); right: 70 (70–73); pelvic radials, left: 3 + 16 (3 + 15–3 + 18); right: 3 + 16 (3 + 15–3 + 18).

*Size*: medium-sized skate reaching at least 711 mm TL. Based on the early subadult male paratype MTUF 30754 of 592 mm TL, males mature at around 600 mm TL. Males of up to 477 mm TL are clearly juvenile with claspers reaching just half of the inner margin length of the posterior pelvic lobes. The smallest known specimen is the juvenile female paratype ZMMU P-17803 with a 276 mm TL. The largest known female with a TL of 700 mm is presumably an adult, judging from the maturing size of males.

**Figure 34:**
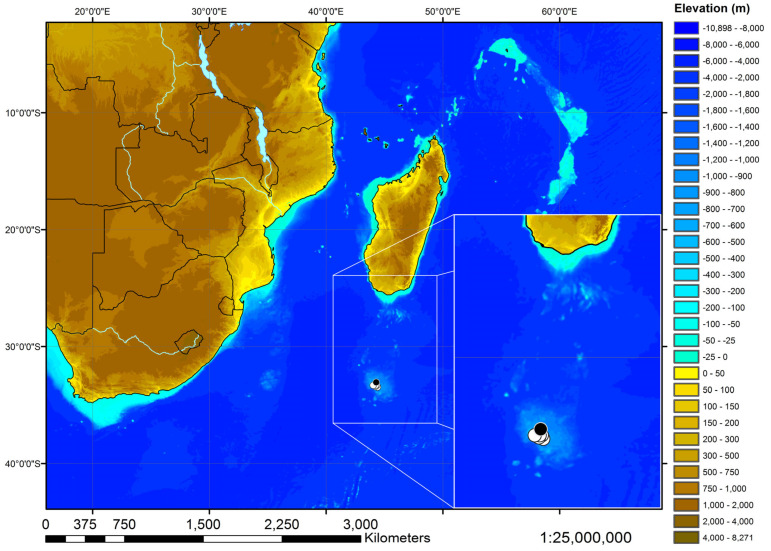
Map of the western Indian Ocean around Madagascar depicting the catch locations of the holotype (black circle) and paratypes (black and white circles) of *Leucoraja longirostris* n. sp. on the southern end of the Madagascar Ridge at Walters Shoals. Inset: close-up of the Madagascar Ridge region.

*Distribution* (Figure 34): the new species is only known from the type specimens, which were all caught on the southern end of the Madagascar Ridge at Walters Shoals at depths of 750–1050 m. The new species occurs much deeper than its western Indian Ocean congeners, which are known only at depths of 480–625 m (*L. compagnoi*), 484 m (*L. elaineae*), and 73–517 m (*L. wallacei*), respectively [1,42]. Furthermore, the new species is the only species of *Leucoraja* in the western Indian Ocean known from seamounts or ridges, whereas its congeners in the area are only known from the outer continental shelf and upper continental slope.

*Etymology*: the species name (lat. *longus* = long, lat. *rostrum* = stiff snout) refers to the remarkably long and pointed snout for a *Leucoraja* species.

*Remarks*: *Leucoraja* comprises 15 valid species. A list of all valid species, including information about species authorships, common names, maximum sizes, as well as depths, and geographic distributions can be found in Table 6. The list has been updated from Weigmann [1].

**Table 6:** Checklist of the 15 valid species of *Leucoraja*.

	Species Authorship	Common Name	Maximum Size	Depth Distribution	Geographic Distribution	Remarks
*L. circularis*	(Couch, 1838)	Sandy ray	1200 mm	10–800 m	NEA	
*L. compagnoi*	(Stehmann, 1995)	Tigertail skate	>517 mm	480–625 m	WIO, SEA	
*L. elaineae*	Ebert and Leslie, 2019	Elaine’s skate	>330 mm	484 m	WIO	
*L. erinacea*	(Mitchill, 1825)	Little skate/Hedgehog skate	620 mm	10–914 m	NWA	
*L. fullonica*	(Linnaeus, 1758)	Shagreen ray	1200 mm	30–600 m	NEA	Possibly occurs down to 800 m depth [43].
*L. garmani*	(Whitley, 1939)	Freckled skate/Rosette skate	570 mm	33–530 m	NWA	*L. garmani* contains two valid subspecies: *L. garmani caribbaea* (McEachran, 1977) and *L. garmani virginica* (McEachran, 1977) [44,45,46].
*L. lentiginosa*	(Bigelow and Schroeder, 1951)	Speckled skate	440 mm	53–588 m	NWA	
*L. leucosticta*	(Stehmann, 1971)	White-dappled skate	800 mm	70–704 m	NEA	Maximum depth of 600 m according to Stehmann [47] but one specimen from 704 m depth is present in the collection of the Fauna Marina del C. O. de Málaga.
***L. longirostris*** **n. sp.**	**Weigmann, Stehmann, Séret and Ishihara**	**Brown longnose skate**	**711 mm**	**750–1050 m**	**WIO**	
*L. melitensis*	(Clark, 1926)	Maltese ray	500 mm	60–800 m	NEA (Mediterranean)	
*L. naevus*	(Müller and Henle, 1841)	Cuckoo ray	810 mm	12–900 m	NEA	Sizes of up to 1060 mm, occasionally reported for specimens from the North Sea [48], are probably erroneous.
*L. ocellata*	(Mitchill, 1815)	Winter skate	1130 mm	4–723 m	NWA	Much larger specimens of up to 1500 mm were captured by the Fisheries Research Board of Canada off central and northern Nova Scotia from 1960 to 1971 [49].
*L. pristispina*	Last, Stehmann and Séret, 2008	Sawback skate	401 mm	202–504 m	EIO	
*L. wallacei*	(Hulley, 1970)	Yellow-spotted skate	963 mm	73–517 m	WIO, SEA	
*L. yucatanensis*	(Bigelow and Schroeder, 1950)	Yucatan skate/Yucatan white skate	300 mm	192–457 m	NWA	

Abbreviations: EIO = eastern Indian Ocean, NEA = northeastern Atlantic Ocean, NEA (Mediterranean) = Mediterranean Sea, NWA = northwestern Atlantic Ocean, SEA = southeastern Atlantic Ocean, WIO = western Indian Ocean.

## 4. Discussion

The new species differs from all congeners in the remarkably long and acutely angled snout (horizontal preorbital length 17.2–22.6% TL vs. 8.5–11.9% TL and 4.2–6.1 vs. 1.7–3.5 times the orbit length, snout angle 65–85° vs. 90–150°). Furthermore, it is apparently endemic to the Madagascar Ridge, distant from the known distribution areas of all congeners. In addition to *L. fullonica* and *L. pristispina*, *L. longirostris* n. sp. is also the only species with plain dorsal coloration. Furthermore, the new species is the only *Leucoraja* species with an external clasper component dike and, besides *L. wallacei*, the only one with four dt cartilages. The shape of the at1 with four tips is also unique within the genus. A detailed comparison of the external clasper characters of all species of the genus with known clasper features can be found in Table 7, and a detailed comparison of skeletal clasper characters can be found in Table 8.

Considering the snout length and dorsal coloration, the new species resembles *L. fullonica* from the northeastern Atlantic and *L. pristispina* from off western Australia.

*L. longirostris* n. sp. differs from *L. fullonica* in having a much longer snout (preorbital length 17.2–22.6% vs. 10.4–11.8% TL and 4.2–6.1 vs. 2.3–2.6 times the orbit length, preoral length 18.4–23.5 vs. 10.7–13.2% TL and 2.8–3.6 vs. 1.4–1.8 times the mouth width), a more acutely angled snout (65–85° vs. 90–100°), coloration (dorsal surface plain medium-brown, ventral side plain grayish on the disc and pelvic fins, but with a snout that is dusky grayish vs. a dorsal surface that is plain ashy-gray, and a ventral side that is totally white), the presence (vs. absence) of the clasper component dike, the presence of four (vs. three) dt cartilages, and an at1 cartilage with four (vs. two) tips. The new species is further distinguished from *L. fullonica* in having broadly rounded pectoral apices (vs. angular), fewer tooth rows in the upper jaw (41–49 vs. 58–68), different thorn patterns (pronounced nuchoscapular triangle present vs. absent, midline of thorns on the trunk and tail present at all life stages vs. reduced or disappearing with age), much sparser denticle coverage (dorsal disc largely devoid of denticles, ventrally spinules mostly confined to the snout vs. largely prickly on the dorsal and ventral surfaces), lower numbers of pectoral radials (69–73 vs. 86–88) and trunk (27–30 vs. 34–36), predorsal tail (60–64 vs. 72–78), and total predorsal (88–94 vs. 106–114) vertebrae, smaller maturity and maximum sizes (maturity size of males 60 cm TL vs. 75–81 cm TL, maximum size 71 cm TL vs. 120 cm TL), deeper occurrence (750–1050 m vs. 30–600 m depth), allopatric distribution (Madagascar Ridge vs. northeastern Atlantic from Norway and Iceland to Morocco including the Mediterranean Sea), as well as numerous morphometric measurements and skeletal features.

*L. longirostris* n. sp. differs from *L. pristispina* in having a distinctly longer snout (preorbital length 17.2–22.6% vs. 9.5–11.9% TL and 4.2–6.1 vs. 2.8–3.5 times the orbit length, preoral length 18.4–23.5 vs. 13.2–13.9% TL and 2.8–3.6 vs. 1.4–1.7 times the mouth width), a more acutely angled snout (65–85° vs. 90–104°), and the presence (vs. absence) of the clasper component dike. The new species is further distinguished from *L. pristispina* in the denticle coverage (the dorsal disc is largely devoid of denticles, ventrally spinules are present at least on the snout vs. dorsal disc very spiny, almost completely covered with fine denticles, and ventrally naked), more pronounced lateral tail folds (extending along the posterior 58.1–72.0% of the tail vs. confined to the posterior half), pelvic fins with 3 + 15–3 + 18 vs. 2 + 18–2 + 22 radials, higher total vertebrae counts (127–136 vs. 121–127), larger maturity and maximum sizes (the maturity size of males is 60 cm TL vs. 33–34 cm TL, the maximum size is 71 cm TL vs. 40 cm TL), deeper occurrence (750–1050 m vs. 202–504 m depth), allopatric distribution (Madagascar Ridge vs. off western Australia), as well as numerous morphometric measurements and skeletal features.

Geographically, the new species occurs closest to *L. compagnoi*, *L. elaineae*, and *L. wallacei*, the only other species of *Leucoraja* known from the (south)western Indian Ocean. *Leucoraja compagnoi* and *L. wallacei* are the only two species of the genus, except for the more northerly distributed *L. leucosticta*, also known from the southeastern Atlantic Ocean.

Compared to *L. compagnoi*, *L. longirostris* n. sp. has a much longer snout (preorbital length 17.2–22.6% vs. 11.0% TL and 4.2–6.1 vs. 2.4 times the orbit length, preoral length 18.4–23.5 vs. 10.8% TL and 2.8–3.6 vs. 1.4 times the mouth width), a more acutely angled snout (65–85° vs. 111°), a differing coloration (tail with plain dorsal coloration vs. tail with broad dark brownish cross-bands), more pronounced lateral tail folds (extending along the posterior 58.1–72.0% of the tail vs. confined to the posterior third), anterior pelvic-fin lobes that are much shorter than the posterior ones (vs. both lobes being nearly equal in length), more tooth rows in the upper jaw (41–49 vs. 38), deeper occurrence (750–1050 m vs. 480–625 m depth), allopatric distribution (Madagascar Ridge vs. off South Africa), as well as numerous morphometric measurements and skeletal features.

*Leucoraja longirostris* n. sp. can easily be distinguished from *L. elaineae* and *L. wallacei* due to the distinctive coloration of the two latter species consisting of one large prominent ocellus about equal to the orbit diameter on each pectoral fin mid-base (*L. elaineae*) or clusters of bright yellow spots and a dark blotch on the tip of the anterior lobe of the pelvic fin (*L. wallacei*). The new species further differs from *L. elaineae* and *L. wallacei* in having a much longer snout (preorbital length 17.2–22.6% vs. 11.9% vs. 9.6–11.2% TL and 4.2–6.1 vs. 2.2 vs. 2.7–2.8 times the orbit length, preoral length 18.4–23.5 vs. 13.8% vs. 10.2–12.4% TL and 2.8–3.6 vs. 1.5 vs. 1.2–1.6 times the mouth width), a more acutely angled snout (65–85° vs. ~120° [the value of 94.7° given by Ebert and Leslie [42] is erroneous] vs. >110°), the presence (vs. unknown vs. absence) of the clasper component dike, an at1 cartilage with four (vs. unknown vs. two) tips, thorn patterns (pronounced nuchoscapular triangle present vs. weakly pronounced vs. usually incomplete; midline of thorns on trunk and tail present at all life stages vs. weakly pronounced vs. reduced or disappearing with age), a longer rostral shaft (68.5–72.0% vs. 45.9% vs. ~45.4% of total cranium length), fewer tooth rows in the upper jaw (41–49 vs. 57 vs. 59–69), lower predorsal tail vertebrae counts (60–64 vs. 72 [recount of holotype] vs. 64–74), smaller maturity and maximum sizes (maturity size of males 60 cm TL vs. 64–77 cm TL, maximum size 71 cm TL vs. 96 cm TL; both sizes unknown for *L. elaineae*), deeper occurrence (750–1050 m vs. 484 m depth vs. 73–517 m depth), allopatric distribution (Madagascar Ridge vs. off Kenya vs. off Namibia to Mozambique), as well as numerous morphometric measurements and skeletal features. The recently described *L. elaineae*, however, requires further investigation as several differences to *L. wallacei* mentioned by Ebert and Leslie [42] are based on erroneous data for *L. elaineae*, and re-examinations have shown close similarity to *L. wallacei* concerning relevant distinguishing features, particularly snout angle, vertebral counts, and coloration: (1) Ebert and Leslie [42] indicated a snout angle of 94.7° for the holotype and only known specimen of *L. elaineae*. However, remeasurements taken from photographs of the holotype showed a much more obtusely angled snout, with a snout angle of ~120°, which is similar to the snout angle of >110° reported for *L. wallacei*. (2) Ebert and Leslie [42] described a “much higher” predorsal vertebral count for *L. elaineae* (107) as compared to *L. wallacei* (64–74). However, they compared the total predorsal vertebral count of *L. elaineae* (107 according to the original description and 104 based on recounted data) with the predorsal tail vertebral count (64–74) of *L. wallacei*. When comparing the predorsal tail vertebral counts of both species, they actually overlap, with the predorsal tail vertebral count of *L. elaineae* being 72 based on recounted data (75 according to the original description) and that of *L. wallacei* being 64–74. (3) Ebert and Leslie [42] reported a different dorsal coloration for both species. However, high-quality scans of P. A. Hulley’s images of the possibly lost holotype of *L. wallacei*, kindly provided by Mark W. Lisher, indicate a coloration rather similar to that of the holotype of *L. elaineae* and different from the coloration described and shown for *L. wallacei* by Ebert and Leslie [42]. The description of the dorsal coloration of *L. wallacei* provided by Hulley [30] in the original description of the species is also rather similar to that of *L. elaineae*: “Upper surface uniformly brown with scattered, irregular lighter spots. *Naevus*-like ocellus at base of each pectoral.” Considering the issues with these three diagnostic features, a re-examination and further specimens of *L. elaineae* are desirable.

The long snout of the new species might impose the risk of it being confused with long-snouted species of other rajid genera, particularly those of *Dipturus* Rafinesque, 1810, *Okamejei* Ishiyama, 1958, and *Orbiraja* Last, Weigmann and Dumale, 2016, but also *Rostroraja alba* (Lacepède, 1803). However, members of *Dipturus* have a very solid rostral cartilage and an anterior cranial fontanelle lacking a clear-cut anterior limit, almost completely lack thorns on the dorsal disc, and have their mucus pores marked black [4,7]. The majority of the species of *Okamejei* and *Orbiraja* superficially resemble *Leucoraja longirostris* n. sp. with their long and pointed snout, slender tail, thorn pattern, and often reduced dorsal spinulation. Nevertheless, they mostly have a very slender tail, widely separated dorsal fins with thorns in the interspace, an obviously long postdorsal tail section, dorsal color pattern, white ventral surface, and dark-marked ventral pores [4,27,53,54]. Furthermore, the *Okamejei* and *Orbiraja* species have very short rostral appendices [27,55,56] and—at least in *Okamejei*—longitudinally expanded, rectangular scapulocoracoids, e.g., [53,57]. *Rostroraja alba*, which also has a long and pointed snout, is not uncommon in the southwestern Indian Ocean northwards to about 20° S and was reported even from northeast of Madagascar, based on a subadult female [56]. Nevertheless, this species has a *Dipturus*-like massive rostral cartilage (albeit a clear cut anterior cranial fontanelle), a broadly flattened tail with strong lateral and median thorns, and a white underside with broad dark disc margins in juveniles [4,56].

External and skeletal features of the claspers are important characters for the differentiation of species and, particularly, genera of skates [7,9,30,31,53,54,58]. Nevertheless, the interpretation of these features is often difficult, even for the experienced taxonomist. Classical pencil drawings have been considered the gold standard for more than 100 years in elasmobranch taxonomy, clearly outperforming photographs or radiographs, e.g., [7,27,30,31,38,41,50,51,55,58,59,60,61,62]. Recently, digital taxonomic drawings have emerged as an alternative, which has been employed to further simplify the features and, thereby, enable easier interpretation of the drawings, e.g., [9,53,54]. An adequate interpretation of clasper features is particularly desirable in taxa with complex claspers, such as legskates, e.g., [58,60,63], or rajid skates, e.g., [7,9,30,38,50,51,53,54,61]. In order to facilitate the interpretation of the external and skeletal clasper morphology, a new approach was developed herein, in which every single external component and internal cartilage of the clasper was dissected and all cartilages were cleaned step by step. Afterwards, 3D models were created on the computer for each single component. This approach makes it possible to rotate every single external and internal component in all directions, assemble and dissemble the clasper in any way, mask and unmask any features, show individual components with selectable levels of transparency, and spread open and close the clasper as desired. This enables a much easier and much more precise interpretation of every single clasper component, of the entire structure and, in particular, the relationship of external features and skeletal cartilages. Such a tool may help more researchers to interpret and understand the complex external and skeletal clasper structures and make comparisons with other material, whether from museum collections or freshly collected.

**Key to the species of *Leucoraja* in the Indian Ocean:**

1. Dorsal coloration ornamented, or at least tail with dark cross-bands, snout short and obtusely angled (horizontal preorbital length 2.2–2.8 times orbit length, snout angle 110–120°)…………………………………………………………………………………… 2

- Dorsal coloration uniformly brownish or grayish without any pattern, snout moderately long or long and slightly obtusely or acutely angled (horizontal preorbital length 2.8–6.1 times orbit length, snout angle 65–104°)…………………....................... 4

2. Dorsal disc plain colored, tail with broad cross-bands; anterior and posterior pelvic-fin lobes of nearly equal length; dorsal fins confluent; 38 tooth rows in the upper jaw …. *L. compagnoi* (southeastern Atlantic and southwestern Indian Ocean: South Africa)

- Distinctive dorsal coloration of one large prominent ocellus about equal to orbit diameter on each pectoral fin mid-base or clusters of bright yellow spots; anterior pelvic-fin lobes much shorter than posterior lobes; dorsal fins separated by short but distinct interspace; 57–69 tooth rows in the upper jaw ………………………………... 3

3. Dorsal coloration variable, holotype uniformly brown with a few lighter spots and a prominent ocellus at each pectoral fin mid-base, but some specimens with more vivid pattern consisting of clusters of bright yellow spots and a dark blotch on the tip of the anterior lobe of the pelvic fin; tail with 3–4 colorful cross-bands formed by larger rosettes and whorls; ventral surface largely plain whitish; spinules ventrally on snout tip and along the anterior margins of the disc ………………............................ *L. wallacei* (southeastern Atlantic and southwestern Indian Ocean: Namibia to Mozambique)

- Dorsal coloration similar to that of the holotype of *L. wallacei*, consisting of one large prominent ocellus about equal to the orbit diameter on each pectoral fin mid-base; tail without any banding; ventral surface whitish but with brownish disk margins and brownish-gray spots on the tail; ventral surface smooth, lacking any prickles ………...………………………………………. *L. elaineae* (western Indian Ocean: Kenya)

4. Snout moderately long and slightly obtusely angled, preorbital length 2.8–3.5 times orbit length, preoral length 1.4–1.7 times mouth width, snout angle 90–104°, dorsal disc very prickly, almost completely covered with fine denticles, ventrally naked, lateral tail folds confined to posterior half of tail, males mature at 33–34 cm TL, maximum size is 40 cm TL …. *L. pristispina* (southeastern Indian Ocean: western Australia)

- Snout long and acutely angled, preorbital length 4.2–6.1 times orbit length, preoral length 2.8–3.6 times mouth width, snout angle 65–85°, dorsal disc largely devoid of denticles, ventrally spinules at least on the snout, lateral tail folds along posterior 58–72% of tail, males mature at 60 cm TL, maximum size is 71 cm TL ……… *L. longirostris* n. sp. (southwestern Indian Ocean: Madagascar Ridge)

## 5. Conclusions

The conservation status of *Leucoraja* skates reveals that five of the 14 assessed species are threatened, including *L. melitensis* (Critically Endangered), *L. circularis* and *L. ocellata* (Endangered), *L. fullonica* and *L. wallacei* (Vulnerable), while one species (*L. leucosticta*) is Near Threatened, six species (*L. erinacea*, *L. garmani*, *L. lentiginosa*, *L. naevus*, *L. pristispina*, and *L. yucatanensis*) are Least Concern, and two species (*L. compagnoi* and *L. elaineae*) are Data Deficient [64]. The recognition of a new species, *Leucoraja longirostris* n. sp., provides new insights into the morphological variation within the genus *Leucoraja* and constitutes a very unusual and remarkable addition to this skate genus. Nevertheless, the very restricted distribution of the new species raises concerns over its ability to sustain fisheries and it may be susceptible to capture in longline and, particularly, deep-water trawl fisheries. Very little information is available about fisheries operating in the area of the Madagascar Ridge, but this deep-water skate is likely unable to withstand intensive fishing pressure due to its potentially slow life history characteristics and low productivity. Walters Shoals was previously heavily fished, and this pressure may recur in the future. As fisheries targeting Orange Roughy (*Hoplostethus atlanticus* Collett, 1889) and Alfonsino (*Beryx decadactylus* Cuvier, 1829) have typically used mid-water trawls off the bottom, the new species may have a benthic refuge [65]. However, further research is needed investigating its distribution, life history, population size and trends, and threats. This is essential for improved data collection and research, and for more effective conservation and management policy decisions.

## Figures and Tables

**Table 7:** Comparison of external clasper characters of *Leucoraja* spp. (unknown for *L. compagnoi*, *L. elaineae*, *L. melitensis*, and *L. yucatanensis*).

	ps	sh	di	rh	ep	cf	se	sl	st	sk	pt	rl	sp	pe
*L. circularis*	+	+	-	+	-	+	-	+	+	+	+	+	+	-
*L. erinacea*	+?	+	-	+	+	++	+	+	+	+	+	+	-	+
*L. fullonica*	+	+	-	+	-	++	-	+	+	+	+	+	+	-
*L. garmani*	-	+	-	+	-	+	+	+	+	+	?	?	-	+
*L. lentiginosa*	-	+	-	+	-	+	+	+	+	+	?	?	-	+
*L. leucosticta*	+	+	-	+	-	+	+	+	+	+	+	?	+	-
***L. longirostris*** **n. sp.**	-	+	+	+	-	++	-	+	+	+	+	+	-	+
*L. naevus*	+	+	-	+	-	++	-	+	+	+	+*	+	+	-
*L. ocellata*	+	+	-	+	+	++	+	+	+	+	+	+	-	+
*L. pristispina*	-	+	-	+	+	+	-	+	+	+	+	+	-	-
*L. wallacei*	-	+	-	+	-	+	-?	+	+	+	+	+	+	+?

Abbreviations: cf = cleft, di = dike, ep = eperon, pe = pent, ps = pseudosiphon, pt = promontory, rh = rhipidion, rl = roll, se = sentina, sh = shield, sk = spike, sl = slit, sp = spur, st = sentinel. Legend of symbols: + = present/yes; - = absent/no; ++ = two times present; +* = bifurcated; +? = possibly present; -? = possibly absent; ? = unknown. Data sources: data on *L. longirostrsis* n. sp. from the present study, on *L. circularis*, *L. fullonica*, and *L. naevus* from Stehmann [7], on *L. erinacea* and *L. ocellata* from McEachran and Martin [50], on *L. garmani* and *L. lentiginosa* from McEachran [38], on *L. leucosticta* from Stehmann [51], on *L. pristispina* from Last et al. [52], and on *L. wallacei* from Hulley [31].

**Table 8:** Comparison of skeletal clasper characters of *Leucoraja* spp. (unknown for *L. compagnoi*, *L. elaineae*, *L. melitensis*, *L. pristispina*, and *L. yucatanensis*).

	dt1	dt2	dt3	dt4	vt	at1	at2	fc
*L. circularis*	+	+	++	-	+	++	+	?
*L. erinacea*	+	+	+	-	+*	+++	+	?
*L. fullonica*	+	+	++	-	+	++	+	+
*L. garmani*	+	+	+	-	+*	++**	+	?
*L. lentiginosa*	+	+	?	-	+	++**	+	?
*L. leucosticta*	+	+	++	-	+	++	+	?
***L. longirostris* n. sp.**	+	+	+	+	+	++++	+	+
*L. naevus*	+	++	++	-	+	++	+	?
*L. ocellata*	+	+	+	-	+	++	+	?
*L. wallacei*	+	+	++	+	+	++	+	?

Abbreviations: at1 = accessory terminal 1, at2 = accessory terminal 2, dt1 = dorsal terminal 1, dt2 = dorsal terminal 2, dt3 = dorsal terminal 3, dt4 = dorsal terminal 4, fc = fibrocartilage, vt = ventral terminal. Legend of symbols: + = present/yes; - = absent/no; ++ = bifurcated; +++ = trifurcated; ++++ = quadfurcated; ? = unclear; * = short and stout, lacking long proximal branch; ** = distal tip margin serrated. Data sources: data on *L. longirostrsis* n. sp. from the present study, on *L. circularis*, *L. fullonica*, and *L. naevus* from Stehmann [7], on *L. erinacea* and *L. ocellata* from McEachran and Martin [50], on *L. garmani* and *L. lentiginosa* from McEachran [38], on *L. leucosticta* from Stehmann [51], and on *L. wallacei* from Hulley [31].

## Data Availability

The data presented in this study are available in this published article.

## References

[1] Weigmann S. (2016). Annotated checklist of the living sharks, batoids and chimaeras (Chondrichthyes) of the world, with a focus on biogeographical diversity. J. Fish Biol..

[2] Weigmann S. (2017). Reply to Borsa (2017): Comment on ‘Annotated checklist of the living sharks, batoids and chimaeras (Chondrichthyes) of the world, with a focus on biogeographical diversity by Weigmann (2016)’: Reply to borsa’s comment on weigmann (2016). J. Fish Biol..

[3] Last P.R., Weigmann S., Yang L. (2016). Changes to the nomenclature of the skates (Chondrichthyes: Rajiformes). Rays of the World: Supplementary Information.

[4] Last P.R., Séret B., Stehmann M.F.W., Weigmann S., Last P.R., White W.T., Carvalho M.R., de Séret B., Stehmann M.F.W., Naylor G.J.P. (2016). Skates, family *Rajidae*. Rays of the World.

[5] Malm A.W. (1877). Göteborgs Och Bohusläns Fauna.

[6] Jordan D.S. (1919). The Genera of Fishes.

[7] Stehmann M. (1970). Vergleichend morphologische und anatomische Untersuchungen zur Neuordnung der Systematik der nordostatlantischen *Rajidae* (Chondrichthyes, Batoidei). Arch. Fisch..

[8] McEachran J.D., Dunn K.A. (1998). Phylogenetic Analysis of Skates, a Morphologically Conservative Clade of Elasmobranchs (Chondrichthyes: Rajidae). Copeia.

[9] Weigmann S., Stehmann M.F.W., Thiel R. (2014). *Rajella paucispinosa* n. sp., a new deep-water skate (*Elasmobranchii*, *Rajidae*) from the western Indian Ocean off South Mozambique, and a revised generic diagnosis. Zootaxa.

[10] Yoon H.K., Jeong D., Chung I., Jung J.W., Oh M.J., Kim S., Lee Y.-H., Kim C.-G., Hwang S.Y. (2009). Rapid Species Identification of Elasmobranch Fish (Skates and Rays) using Oligonucleotide Microarray. BioChip J..

[11] Pfaff C., Kriwet J., Martin K., Johanson Z. (2018). Ontogenetic development of the otic region in the new model organism, *Leucoraja erinacea* (Chondrichthyes; Rajidae). Earth Environ. Sci. Trans. R. Soc. Edinb..

[12] Gillis J.A., Bennett S., Criswell K.E., Rees J., Sleight V.A., Hirschberger C., Calzarette D., Kerr S., Dasen J. (2022). Big insight from the little skate: *Leucoraja erinacea* as a developmental model system. Current Topics in Developmental Biology.

[13] Yoo D., Park J., Lee C., Song I., Lee Y.H., Yun T., Lee H., Heguy A., Han J.Y., Dasen J.S. (2022). Little skate genome provides insights into genetic programs essential for limb-based locomotion. eLife.

[14] Marlétaz F., De La Calle-Mustienes E., Acemel R.D., Paliou C., Naranjo S., Martínez-García P.M., Cases I., Sleight V.A., Hirschberger C., Marcet-Houben M. (2023). The little skate genome and the evolutionary emergence of wing-like fins. Nature.

[15] Nykänen M., Dillane E., Reid D., Rogan E. (2020). Genetic methods reveal high diversity and no evidence of stock structure among cuckoo rays (*Leucoraja naevus*) in the northern part of Northeast Atlantic. Fish. Res..

[16] Turan C. (2008). Molecular Systematic Analyses of Mediterranean Skates (*Rajiformes*). Turk. J. Zool..

[17] Aschliman N.C., Nishida M., Miya M., Inoue J.G., Rosana K.M., Naylor G.J.P. (2012). Body plan convergence in the evolution of skates and rays (Chondrichthyes: Batoidea). Mol. Phylogenet. Evol..

[18] Naylor G., Caira J., Jensen K., Rosana K., Straube N., Lakner C., Carrier J.C., Musick J.A., Heithaus M.R. (2012). Elasmobranch Phylogeny: A Mitochondrial Estimate Based on 595 Species. Biology of Sharks and Their Relatives.

[19] Naylor G.J.P., Caira J.N., Jensen K., Rosana K.A.M., White W.T., Last P.R. (2012). A DNA sequence–based approach to the identification of shark and ray species and its implications for global elasmobranch diversity and parasitology. Bull. Am. Mus. Nat. Hist..

[20] Chiquillo K.L., Ebert D.A., Slager C.J., Crow K.D. (2014). The secret of the mermaid’s purse: Phylogenetic affinities within the *Rajidae* and the evolution of a novel reproductive strategy in skates. Mol. Phylogenet. Evol..

[21] Lynghammar A., Christiansen J.S., Griffiths A.M., Fevolden S., Hop H., Bakken T. (2014). DNA barcoding of the northern Northeast Atlantic skates (Chondrichthyes, Rajiformes), with remarks on the widely distributed starry ray. Zool. Scr..

[22] Compagno L.J.V., Ebert D.A. (2007). Southern African skate biodiversity and distribution. Environ. Biol. Fishes.

[23] Compagno L.J.V., Stehmann M., Ebert D.A. (1990). *Rhinochimaera africana*, a new longnose chimaera from southern Africa, with comments on the systematics and distribution of the genus *Rhinochimaera* Garman, 1901 (Chondrichthyes, Chimaeriformes, Rhinochimaeridae). S. Afr. J. Mar. Sci..

[24] Fricke R., Eschmeyer W.N. Eschmeyer’s Catalog of Fishes: Guide to Fish Collections. http://researcharchive.calacademy.org/research/ichthyology/catalog/collections.asp.

[25] Bigelow H.B., Schroeder W.C. (1953). Fishes of the Western North Atlantic Part Two: Sawfishes, Guitarfishes, Skates and Rays, Chimaeroids.

[26] Clark R.S. (1926). Rays and skates. A revision of the European species. Fish. Board Scotl. Sci. Investig..

[27] Ishiyama R. (1958). Studies on the rajid fishes (Rajidae) found in the waters around Japan. J. Shimonoseki Coll. Fish..

[28] Hubbs C.L., Ishiyama R. (1968). Methods for the Taxonomic Study and Description of Skates (*Rajidae*). Copeia.

[29] Stehmann M. (1985). Ergebnisse der Forschungsreisen des FFS “Walther Herwig” nach Südamerika. LXIV. *Bathyraja papilionifera* sp. n. (Pisces, Batoidea, Rajidae), eine weitere neue Rochenart aus dem Südwestatlantik vom nordargentinischen Kontinentalabhang. Arch. Fisch..

[30] Hulley P.A. (1970). An investigation of the Rajidae of the west and south coasts of Southern Africa. Ann. S. Afr. Mus..

[31] Hulley P.A. (1972). The origin, interrelationship and distribution of Southern African Rajidae (Chondrichthyes, Batoidei). Ann. S. Afr. Mus..

[32] McEachran J.D., Compagno L.J.V. (1979). A Further Description of *Gurgesiella furvescens* with Comments on the Interrelationships of Gurgesiellidae and Pseudorajidae (Pisces, Rajoidei). Bull. Mar. Sci..

[33] Stehmann M.F.W., Séret B., Costa E.M., Baro J. (2008). *Neoraja iberica* n. sp., a new species of pygmy skate (*Elasmobranchii*, *Rajidae*) from the southern upper slope of the Iberian Peninsula (Eastern North Atlantic). Cybium.

[34] Springer V.G., Garrick J.A.F. (1964). A Survey of Vertebral Numbers in Sharks. Proc. United States Natl. Mus..

[35] Krefft G. (1968). Knorpelfische (Chondrichthyes) aus dem tropischen Ostatlantik. Atlantide Rep..

[36] Amante C., Eakins B.W. (2009). ETOPO1 1 Arc-Minute Global Relief Model: Procedures, Data Sources and Analysis. NOAA Technical Memorandum NESDIS NGDC-24.

[37] Weigmann S., Stehmann M.F.W., Thiel R. (2013). *Planonasus parini* n. g. and n. sp., a new genus and species of false cat sharks (Carchariniformes, Pseudotriakidae) from the deep northwestern Indian Ocean off Socotra Islands. Zootaxa.

[38] McEachran J.D. (1977). Variation in *Raja garmani* and the Status of *Raja lentiginosa* (Pisces: Rajidae). Bull. Mar. Sci..

[39] McEachran J.D., Miyake T., Uyeno T., Arai R., Taniuchi T., Matsuura K. (1986). Interrelationships within a putative monophyletic groups of skates (Chondrichthyes, Rajoidei, Rajini). Indo-Pacific Fish Biology: Proceedings of the Second International Conference on Indo-Pacific Fishes.

[40] Leible M.D., Stehmann M. (1987). First records of *Raja* (*Dipturus*) *trachyderma* Krefft & Stehmann, 1975 from the Southeastern pacific off Chile, with first descriptions of its clasper characters and additional skeletal and morphological details (Pisces, Rajiformes, Rajidae). Stud. Neotrop. Fauna Environ..

[41] Backman G.V. (1913). Die Bauchflosse der Selachier. Erste Abtheilung. Die Bauchflosse der Batoidei. K. Sven. Vetenskabsakad. Handl..

[42] Ebert D.A., Leslie R.W. (2019). *Leucoraja elaineae* sp. nov., a new rough skate (Rajiformes: Rajidae) from the Western Indian Ocean. Zootaxa.

[43] Relini G., Mannini A., de Ranieri S., Bitetto S., Follesa M.C., Gancitano V., Manfredi C., Casciaro L., Sion L. (2010). *Chondrichthyes* caught during the MEDITS surveys in Italian waters. Biol. Mar. Mediterr..

[44] Schmitter-Soto J.J., Vásquez-Yeomans L., Aguilar-Perera A., Curiel-Mondragón C., Caballero-Vázquez J.A. (2000). Lista de peces marinos del Caribe mexicano. An. Inst. Biología. Ser. Zool..

[45] McEachran J.D., Collette B.B., Klein-MacPhee G. (2002). Skates. Family Rajidae. Bigelow and Schroeder’s Fishes of the Gulf of Maine.

[46] Moore J.A., Hartel K.E., Craddock J.E., Galbraith J.K. (2003). An Annotated List of Deepwater Fishes from off the New England Region, with New Area Records. Northeast. Natl..

[47] Stehmann M. (1995). First and new records of skates (Chondrichthyes, Rajiformes, Rajidae) from the West African continental slope (Morocco to South Africa), with descriptions of two new species. Arch. Fish. Mar. Res..

[48] ICES ICES Database on Trawl Surveys (DATRAS). https://datras.ices.dk.

[49] McEachran J.D., Martin C.O. (1977). Possible occurrence of character displacement in the sympatric skates *Raja erinacea* and *R. ocellata* (Pisces: Rajidae). Environ. Biol. Fish..

[50] McEachran J.D., Martin C.O. (1978). Interrelationships and Subgeneric Classification of *Raja erinacea* and *R. ocellata* Based on Claspers, Neurocrania and Pelvic Girdles (Pisces: Rajidae). Copeia.

[51] Stehmann M. (1971). *Raja* (*Leucoraja*) *leucosticta* spec. nov. (Pisces, Batoidei, Rajidae), eine neue Rochenart aus dem Seegebiet des tropischen Westafrika; gleichzeitig zur Frage des Vorkommens von *Raja ackleyi* Garman, 1881, im mittleren Ostatlantik. Arch. Fisch..

[52] Last P.R., Stehmann M., Séret B. (2008). *Leucoraja pristispina* sp. nov., a new deepwater skate from Western Australia. CSIRO Mar. Atmos. Res. Pap..

[53] Weigmann S., Stehmann M.F.W., Thiel R. (2015). *Okamejei ornata* n. sp., a new deep-water skate (*Elasmobranchii*, *Rajidae*) from the northwestern Indian Ocean off Socotra Islands. Deep Sea Res. II Top. Stud. Oceanogr..

[54] Last P.R., Weigmann S., Dumale D. (2016). A new skate genus *Orbiraja* (*Rajiformes*: *Rajidae*) from the Indo-West Pacific. Zootaxa.

[55] Ishiyama R. (1967). Fauna Japonica/Rajidae (Pisces).

[56] Stehmann M. (1976). Revision der Rajoiden-Arten des nördlichen Indischen Ozean und Indopazifik (Elasmobranchii, Batoidea, *Rajiformes*). Beaufortia.

[57] Ishihara H., Ishiyama R., Uyeno T., Arai R., Taniuchi T., Matsuura K. (1986). Systematics and Distribution of the Skates of the North Pacific (Chondrichthyes, Rajoidei). Indo-Pacific Fish Biology: Proceedings of the Second International Conference on Indo-Pacific Fishes.

[58] Weigmann S., Stehmann M.F.W., Thiel R. (2014). Complementary redescription of *Anacanthobatis ori* (Wallace, 1967) and its assignment to *Indobatis* n. g. (Elasmobranchii, Anacanthobatidae), with comments on other legskates. Zootaxa.

[59] Leigh-Sharpe W.H. (1924). The comparative morphology of the secondary sexual characters of elasmobranch fishes. The claspers, clasper siphons, and clasper glands. Memoir VII. J. Morphol..

[60] Hulley P.A. (1973). Interrelationships within the Anacanthobatidae (Chondrichthyes, Rajoidea), with a description of the lectotype of *Anacanthobatis marmoratus* von Bonde & Swart, 1923. Ann. S. Afr. Mus..

[61] Stehmann M. (1978). *Raja* „*bathyphila*“, eine Doppelart des Subgenus *Rajella*: Wiederbeschreibung von *R. bathyphila* Holt & Byrne, 1908 und *Raja bigelowi* spec. nov. (Pisces, Rajiformes, Rajidae). Arch. Fisch..

[62] Stehmann M.F.W. (2012). Complementary redescription of *Raja lintea* Fries, 1839 (Elasmobranchii, Rajidae) and its revised generic assignment. Zootaxa.

[63] Stehmann M.F.W., Weigmann S. (2016). A new deepwater legskate, *Sinobatis kotlyari* n. sp. (Rajiformes, Anacanthobatidae) from the southeastern Indian Ocean on Broken Ridge. Zootaxa.

[64] IUCN (2023). The IUCN Red List of Threatened Species.

[65] Pollom R., Bennett R., Da Silva C., Ebert D.A., Gledhill K., Leslie R., McCord M.E., Weigmann S., Winker H. (2019). *Bythaelurus* *bachi*. The IUCN Red List of Threatened Species.

